# UV-B induced flavonoid accumulation and related gene expression in blue- grained wheat at different periods of time

**DOI:** 10.3389/fpls.2024.1520543

**Published:** 2024-12-16

**Authors:** Li Li, Guofei Jiang, Hanxue Li, Junna Liu, Ping Zhang, Qianchao Wang, Liubin Huang, Shan Zhang, Xuqin Wang, Lingyuan Zhang, Yutao Bai, Peng Qin

**Affiliations:** College of Agronomy and Biotechnology, Yunnan Agricultural University, Kunming, China

**Keywords:** blue-grained wheat, UV-B, flavonoids, transcriptome, F3H, FLS

## Abstract

**Introduction:**

UV-B can be used as an additional technique for nutrient accumulation in blue-grained wheat, which has special nutritional properties due to its blue starch layer. The concentration of flavonoids in blue-grained wheat under UV-B irradiation is extremely important for further investigation and exploitation of the nutritional properties of blue-grained wheat.

**Methods:**

This investigation focuses on the expression of flavonoids and associated genes in blue-grained wheat using transcriptomic and metabolomic analyzes.

**Results:**

The metabolome revealed 1846 compounds and 340 flavonoids after UV-B irradiation. Under UV-B irradiation, the amount of flavonoid metabolites decreased over time, but flavones and flavanols increased, and flavones and flavanols were more diverse and abundant. The content of some flavonoids of blue-grain wheat in period 2 was significantly higher under UV-B irradiation than its check and other periods of different treatments. There are 42344 differentially expressed genes identified from transcriptomic analysis, including 151 genes associated with the flavonoid pathway. The genes for the enzymes *FLS*, *ANR*, *HCT*, *CYP75A* and *CYP73A* are more abundant, with *F3H* and *FLS* showing higher expression levels.

**Discussion:**

The expression of these genes decreased after early UV-B irradiation, but increased later. In the joint WGCNA study of the two groups, the *FLS* enzyme gene *LOC123125079* plays an important role in the response of blue-grained wheat to UV-B irradiation. Our findings help to identify essential genes and processes that allow blue-grained wheat to respond appropriately to UV-B irradiation, which is critical for the accumulation of flavonoids and other bioactive compounds in colored wheat, maximising its nutritional properties.

## 1 Introduction

Light plays a crucial role in regulating plant growth and development (Yadav et al., 2020), while the plant’s response to light exhibits complex and dynamic characteristics. As the amount of ultraviolet (UV) irradiation reaching the Earth increases in response to environmental change, which may have negative consequences on the development of biodiversity and the degradation of ecosystems (Bernhard et al., 2020). Of these, ultraviolet B (UV-B) is the main UV irradiation that reaches the Earth, and increased UV-B irradiation affects plant growth and development (Barnes et al., 2019). In recent years, there has been a huge increase in research into the perception, signaling and response of plants to UV irradiation. Wheat (*Triticum aestivum* L.) is an important staple crop grown throughout the world. Colored wheat is beneficial to human health due to its unique nutritional properties in grain color, and consumers may prefer it as a raw material for functional foods (Chen et al., 2020). Currently, most research focuses on plant physiological and morphological characteristics, with little attention paid to post-flowering grain quality development. Therefore, investigating the effect of post-flowering UV-B irradiation on the quality development of colored wheat grains is an important research topic.

Several studies have shown that UV-B environment affects morphological, physiological and molecular levels of photosynthetic plants (El-Sheekh et al., 2021). Under UV-B irradiation, plants respond to UV-B stress by altering morphological traits and delaying growth, such as by hypocotyl elongation, cuticle thickening, inducing stomatal closure, and leaf curling (Jenkins, 2017; Tossi et al., 2014; Robson et al., 2015; Yadav et al., 2020). Physiologically, plants adapt to UV-B irradiation conditions by increasing antioxidant enzyme activity, proline content, protein concentration, cyclobutane pyrimidine dimer (CPD) photolyase, phenylalanine ammonia-lyase (*PAL*) activity, and flavonoid content, while decreasing chlorophyll content, net photosynthetic rate, stomatal conductance, and transpiration rate (Dwivedi and Ahmad, 2023; Mmbando et al., 2023; Chu et al., 2022). At the molecular level, UV-B irradiation has an effect on primary and secondary metabolism. Under short-term UV-B irradiation, the primary metabolism and growth of plants are indirectly affected by DNA photodimer accumulation and DNA damage and repair (Biever et al., 2014; Yadav et al., 2020). Primary metabolites induce cells to promote the subsequent production of secondary metabolites that absorb UV-B, such as changes in plant carbohydrate content (Ghisi et al., 2002). In addition, UV-B-mediated responses are highly dependent on phytohormones, generally inhibition of gibberellic acid (GA) synthesis and accumulation of abscisic acid (ABA) and jasmonic acid (JA) to enhance plant UV-B tolerance (Yadav et al., 2020, 2020).

UV-B environment promotes flavonoid accumulation in plants, which have a wide range of biological activities that are beneficial to human health (Tungmunnithum et al., 2018), and thus UV-B can be used as an eco-friendly complementary strategy to improve the nutritional properties of crops. Due to their immobility, plants have developed adaptive systems to overcome UV-B irradiation (Chung et al., 2022) with changes in the transcript levels of genes encoding enzymes corresponding to the flavonoid biosynthesis pathway (Hectors et al., 2014), which result in the accumulation of flavonoids for defense against UV-B irradiation (Xie et al., 2022; Shamala et al., 2020; Zeng et al., 2020). In summary, plants undergo extensive metabolic changes in response to UV-B, enabling them to modulate their physiological state and cope with the stress imposed by these wavelengths. The use of UV-B as an inducer of specific metabolites may be a key strategy for improving the nutritional quality of economically important food crops.

Currently, the application of UV-B irradiation to supplement visible light to increase bioactive compounds is increasing. The effects of UV-B irradiation on plants are geographically related to species, latitude, and elevation, respectively, with plants at higher latitudes and lower elevations being more sensitive to UV-B irradiation than plants at lower latitudes and higher elevations (Baroniya et al., 2013; Teramura et al., 1991). Many studies have been conducted on the effects of UV irradiation on photosynthetic organisms, however little is known about the mechanisms by which plants ameliorate the deleterious effects of sustained UV-B irradiation, and research on the effects of UV-B irradiation on colored wheat kernels is still limited, and in particular the mechanisms by which ambient UV-B regulates the enrichment of flavonoids in wheat are still unclear. Therefore, we regulated wheat grain quality by modulating the metabolic flux of flavonoid biosynthesis. This study not only provides a feasible and simple UV-B supplementation strategy to regulate flavonoid biosynthesis in wheat grains, but also contributes to the understanding of photobiological responses associated with secondary metabolism and provides a comprehensive understanding of how wheat grain adapts to environmental stresses in an era of climate change. Our study focuses on the post-flowering response to UV-B in blue-grained wheat, when the morphology of the wheat plant is basically established, and therefore we focus on the metabolite level and transcriptome level to analyze the effect of UV-B on the formation of seed quality in blue-grained wheat.

## 2 Materials and methods

### 2.1 Wheat material and UV-B treatment

**Figure 1:**
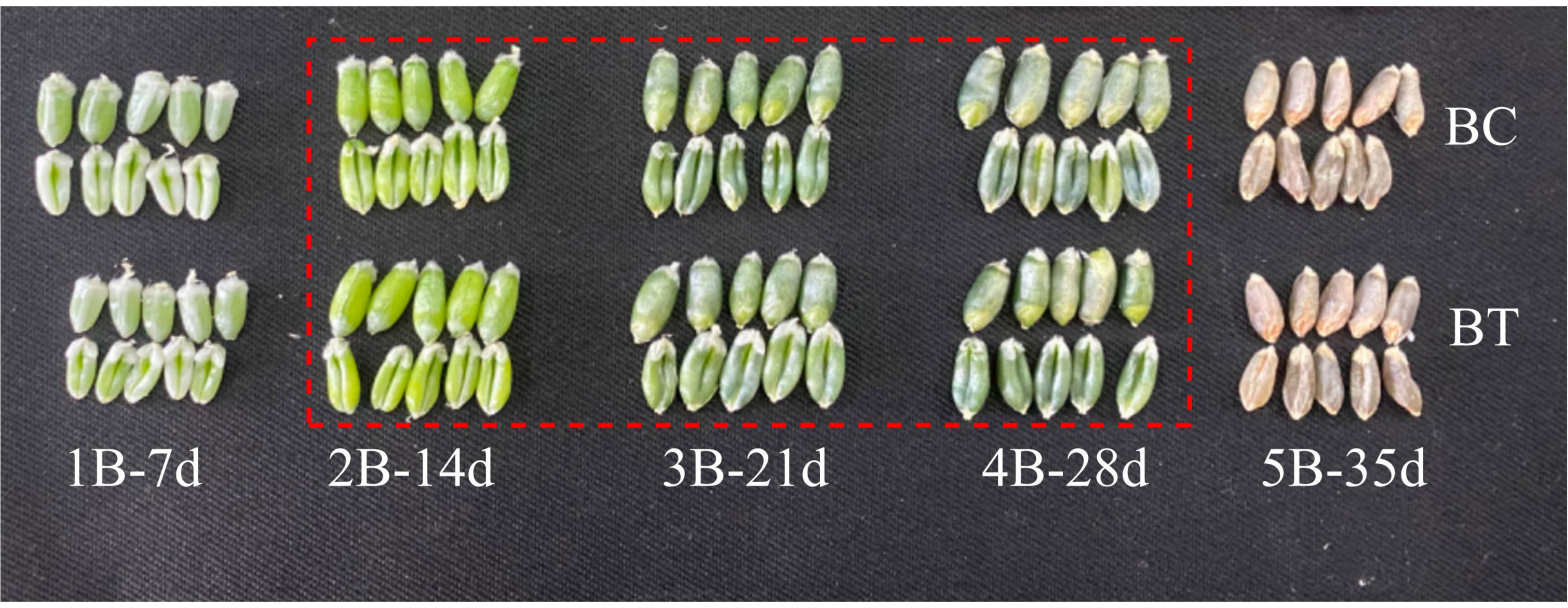
Blue-grained wheat flower after UV-B treatment - control seed image. BC is the control group, and BT is the UV-B treatment. Samples were taken every week after flowering, with the first week’s sample labelled as 1B, and so on, for a total of 5 weeks of sampling until the seeds were mature.

The material used in this experiment was our own blue-grained wheat high-generation line (F8) ‘Dianmai 20-8’, which is a cross between Dianmai 16 and AS905001. The material was planted at the Modernisation Base of Yunnan Agricultural University, Xundian County, Kunming City, Yunnan Province, China. Dianmai 20-8 was sown in October 2022 and harvested in May 2023, with a growth cycle of 8 months, starting to spike and flower in April, and maturing after completing irrigation in about a month of flowering. The average temperature required for flowering is 10°C-29°C and the average humidity is 60%-80%. The material was planted in glass greenhouse strip pots, the planting soil was a mixture of loess and humus 8:2, the soil was loosened and fertilized before planting, and watered every day for a week after sowing, and watered and fertilized as appropriate after a week when the basic emergence of the seedling was complete according to the soil moisture and the growth of the seedling. The methods employed for planting the wheat, as well as the conditions pertaining to water and fertilizer management, were maintained throughout the experiment. Plants exhibiting uniform growth and height patterns were selected for labeling and subjected to UV-B treatment from the first day of flowering until the maturation of the wheat grain. This treatment was administered daily between the hours of 7:00 and 19:00 for a duration of 12 hours, with a corresponding non-UV-B irradiation control group (UV-B treatments: BT, non-UV-B control: BC). The artificial UV-B irradiation source consisted of a single UV-B rod (spectral λmax = 313 nm; TL 40W/01 RS; kpc), which was fixedly mounted 10 cm above the spike of the wheat plant (0.74 mW/cm²). The height of the lamp holder could be adjusted according to the height of the wheat plant. On the first day of flowering, wheat plants with similar agronomic traits and growth were selected for labelling and UV-B treatment, with corresponding controls. Samples were collected weekly from the first day of flowering to maturity, and all samples were taken from the middle of the spike, resulting in a total of five different periods of wheat sampling. According to the degree of color development in blue-grained wheat, the color development started to be obvious in the 4th period (4B-28d), and in order to study the dynamic formation of its quality, the period of the most obvious color development and the first two periods, i.e., the 2nd period (2B-14d), the 3rd period (3B-21d) and the 4th period (4B-28d), in a total of three periods, were studied in depth (**Figure 1**).

### 2.2 Metabolomic profiling and analysis of blue-grained wheat under UV-B irradiation

Metabolomics analysis comprises two principal components: metabolomics experiments and data analysis. The data analysis primarily entails differential metabolite screening and metabolic pathway analysis, with a particular focus on a comprehensive examination of the flavonoid biosynthesis pathway in this experiment. The entire experimental process involves the separation of substances, qualitative and quantitative analysis, and identification using ultra-high-performance liquid chromatography-tandem mass spectrometry (UPLC-MS/MS). A total of 18 biological samples were obtained from the UV-B-irradiation and control groups at three time points. Metabolomic data was then obtained, followed by metabolite identification, sample data quality control analysis, differential metabolite screening, and functional prediction and analysis of the metabolites in the samples. The differential metabolites were selected based on the following criteria: fold change ≥ 2, fold change ≤ 0.5, and VIP > 1. Further details regarding the preparation and extraction of the metabolite samples can be found in the publications by Li et al. (2021, 2023). Wheat grain samples were vacuum freeze-dried, crushed using a mixer for 1.5 min to powder form, then 70% aqueous methanol solution was immediately added to the powdered wheat and placed in a refrigerator at 4°C overnight before centrifuging the extracted supernatant for analysis by ultra performance liquid chromatography mass spectrometry (UPLC-MS/MS). Chromatographic separations were carried out on an Agilent SB-C18 column (1.8 µm, 2.1 mm*100 mm) at 40°C with a mobile phase consisting of 0.1% formic acid in pure water and acetonitrile. The mass spectrometry conditions were electrospray ionization (ESI) at 550°C, mass spectrometry voltage at 5500 V, curtain gas (CUR) at 30 psi, and collision-activated dissociation (CAD) parameter set to high. Metabolite identification annotation was based on the database MWDB (Wuhan Metavir Biotechnology Co., Ltd., Wuhan, China, http://www.metware.cn/), substance characterization was performed based on secondary spectrum information, and metabolite quantification was performed by triple reaction monitoring (MRM) mode of triple quadrupole mass spectrometry (TQMS), and the peaks detected in different samples of each metabolite was corrected for the mass spectral peaks detected in different samples to ensure the accuracy of the characterization and quantification. The analysis removes isotopic signals, repetitive signals containing K^+^, Na^+^, NH_4_^+^, and repetitive signals from fragment ions that are themselves other substances of larger molecular weights. The raw data was subjected to metabolite annotation following the extraction and correction of mass spectral peaks. It is necessary to filter out low-quality data through data quality control for statistical analysis. The results of the metabolite data are evaluated through qualitative and quantitative analysis of the metabolites, sample quality control analysis, principal component analysis, and cluster analysis. Finally, a differential gene clustering heatmap and metabolite pathway analysis is conducted on comparison groups, with a focus on in-depth analysis of metabolites in the flavonoid synthesis pathway.

### 2.3 Transcriptome sequencing of blue-grained wheat under UV-B irradiation

The process of transcriptome sequencing is comprised of four principal stages. The process of transcriptome sequencing is comprised of four principal stages: RNA extraction, RNA detection, library construction, and sequencing. In this experiment, a total of 18 wheat grain biological samples were subjected to sequencing and subsequent analysis. Initially, total RNA was extracted using RNA simple total RNA kit (Tiangen biotech, Beijing, China) and the integrity, concentration, purity, and contamination were tested to ensure the quality of the RNA for subsequent library construction. The mRNA enriched from total RNA was used as a template for the synthesis and purification of double-stranded cDNA (NEBNextRUltraTMRNA Library Prep Kit, NEB, USA). First strand cDNA was synthesized using random hexamer primer and M-MuLVReverse Transcriptase(RNase H-). Second strand cDNA synthesis was subsequently performed using DNA Polymerase l and RNase H. Remaining overhangs were converted into blunt ends via exonuclease/polymerase activities. After adenylation of 3’ ends of DNA fragments, NEBNext Adaptor with hairpin loop structure were ligated to prepare for hybridization. In order to select cDNA fragments of preferentially 250~300 bp in length, the library fragments were purified with AMPure XP system (Beckman Coulter, Beverly, USA). Then 3 ul USER Enzyme (NEB, USA) was used with size-selected, adaptor-ligated cDNAat 37C for 15 min followed by 5 min at 95C before PCR. Then PCR was performed with Phusion HighFidelity DNA polymerase, Universal PCR primers and Index (X) Primer. At last, PCR products were purified (AMPure XPsystem) and library quality was assessed on the Agilent Bioanalyzer 2100 system. Only once the test results meet the requisite standards may sequencing be performed on the machine. Once the library inspection has been deemed satisfactory, the different libraries are pooled according to the target offline data volume. The Illumina platform was used for sequencing.

### 2.4 Bioinformatics analysis of the transcriptome under UV-B irradiation and validation of quantitative real-time PCR

The raw data must be filtered to obtain clean data, aligned with the wheat reference genome to obtain mapped data, and subjected to structural level analysis. This may include alternative splicing analysis, novel gene discovery, and gene structure optimization based on the alignment results. Expression level analysis may also be conducted, including differential expression analysis, functional annotation of differentially expressed genes, and functional enrichment based on the expression levels of genes in different sample groups. This study employed Fragments Per Kilobase of transcript per Million fragments mapped (FPKM) as a metric to assess transcript or gene expression levels. The differential genes were selected based on a log2 fold change threshold of ≥1 and an False Discovery Rate (FDR) of <0.05. Functional annotations and enrichment analyzes were conducted on differentially expressed genes, including KEGG, GO, KOG, NR, Pfam, SwissProt, TF, and Trembl. The focus was on the flavonoid biosynthesis pathways in the KEGG database. To provide a comprehensive analysis, gene set enrichment analysis (GSEA) was performed on genes with insignificant differential expression but significant biological relevance. The differentially expressed genes that were enriched in the flavonoid synthesis pathway were subjected to further expression profiling analysis. To verify the reliability of the transcriptome sequencing results, all samples were subjected to qRT-PCR (Applied Biosystems™7500, Thermo Fisher Scientific, Massachusetts, USA) in three biological replicates. ATP-dependent 26S proteasome regulatory subunit (26S) was used as an internal reference gene for the assay, and gene primers were designed in Beacon Designer 8.0 (https://www.premierbiosoft.com/molecular_beacons/overview.html) (**Supplementary Table 1**). Relative gene expression levels were calculated using the 2^-ΔΔCT^ method (Livak and Schmittgen, 2001).

### 2.5 Bionomics combined with weighted correlation network analysis to analyze the biosynthesis pathway of flavonoids in blue-grained wheat under UV-B irradiation

The joint analysis of metabolome and transcriptome data primarily comprises KEGG functional enrichment analysis, nine quadrant expression trend analysis, and expression correlation network association analysis. This study is focused on the Ko00941 flavonoid biosynthesis pathway. The content of flavonoid secondary substance classifications (flavones, flavonols, flavanones, flavanols, anthocyanidins, flavanonols, chalcones, aurones, isoflavones, other flavonoids) in the metabolome is employed as a trait basis for mining WGCNA, in conjunction with all genes annotated to the flavonoid biosynthesis pathway for WGCNA data mining. Prior to commencing the WGCNA analysis, it is necessary to filter the input FPKM expression file using the varFilter function from the gene filter package in R language. This is done in order to remove genes with low expression levels across all samples and genes with stable expression levels across all samples. This process serves to enhance the accuracy of network construction. The WGCNA analysis identified gene modules that were highly correlated with traits. The core genes with high connectivity were imported into Cytoscape software to construct a protein-protein interaction network, analyze protein interactions, and select core genes. Finally, both differential metabolites and differential genes were mapped to the flavonoid synthesis pathway in order to analyze the impact of UV-B irradiation on flavonoid synthesis in wheat grains at different stages.

### 2.6 Statistical analysis

In this experiment, all experimental data, including those from the same period and treatment, were derived from three biological replicates. Univariate statistical analysis methods included hypothesis testing and fold change (FC) analysis. Multivariate statistical analysis methods encompass principal component analysis (PCA) and orthogonal partial least squares discriminant analysis (OPLS-DA), among others. Unsupervised PCA (principal component analysis) was performed by statistics function prcomp within R (www.r-project.org). The data was unit variance scaled before unsupervised PCA. A heatmap is generated using the R software ComplexHeatmap package. For two-group analysis, differential metabolites were determined by VIP (VIP > 1) and absolute Log2FC (|Log2FC| ≥ 1.0). VIP values were extracted from OPLS-DA result, which also contain score plots and permutation plots, was generated using R package MetaboAnalystR. The data was log transform (log2) and mean centering before OPLS-DA. In order to avoid overfitting, a permutation test (200 permutations) was performed. Use feature Counts to calculate the gene alignment, and then calculate the FPKM of each gene based on the gene length. DESeq2 was used to analyze the differential expression between the two groups, and the P value was corrected using the Benjamini & Hochberg method. The corrected P value and |log2 fold change| are used as the threshold for significant difference Expression. Finally, use Cytoscape to draw a network diagram.

## 3 Results and analysis

### 3.1 Overall metabolite profile and differential metabolite analysis of blue-grained wheat in response to UV-B

**Figure 2:**
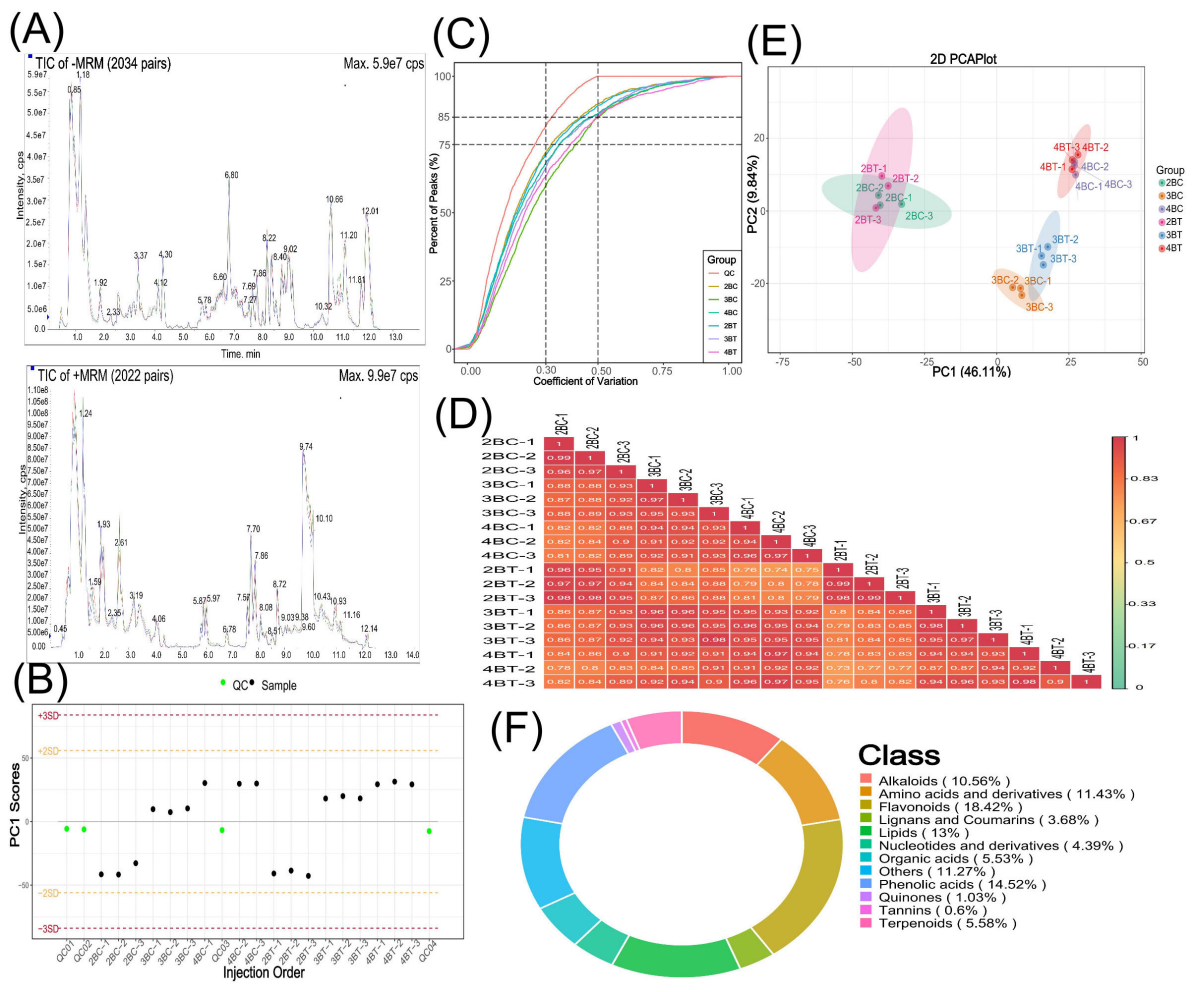
Metabolite overall profile diagram. **(A)** TIC overlap Map of quality control samples by mass spectrometry detection;the abscissa represents the retention time (min) of metabolite detection, and the ordinate represents the intensity of the ion current (cps: count per second); **(B)** Overall sample PC1 control chart. The horizontal coordinate of the graph is the order of sample testing, the vertical coordinate reflects the PC1 value, and the yellow and red lines define a range of plus or minus 2 and 3 standard deviations, respectively. The green dots represent quality control QC samples and the black dots represent test samples; **(C)** CV distribution graph for each group of samples. The horizontal coordinate represents the CV value, the vertical coordinate represents the proportion of the number of substances smaller than the corresponding CV value to the total number of substances, different colors represent different group samples, and QC is the quality control sample, in which the two reference lines perpendicular to the X-axis correspond to the CV value of 0.3 and 0.5, and the two reference lines parallel to the X-axis correspond to the number of substances accounting for 75% and 85% of the total number of substances; **(D)** Correlation plot between samples. The vertical and diagonal lines represent the sample names of the different samples, and different colors represent different Pearson correlation coefficients, with redder colors representing stronger positive correlations, greener colors representing weaker correlations, and bluer colors representing stronger negative correlations, and the correlation coefficient magnitudes of the two samples are marked in the boxes; **(E)** Principal Component Analysis (PCA) plot for the overall sample. pc1 represents the first principal component, pc2 represents the second principal component, pc3 represents the third principal component, and the percentage represents the rate at which that principal component explains the data set; each point in the plot represents a sample, and samples in the same group are represented using the same color, Group is Group; **(F)** Metabolite categories make up the ring. Each color represents a metabolite category and the area of the color block indicates the proportion of that category.

**Figure 3:**
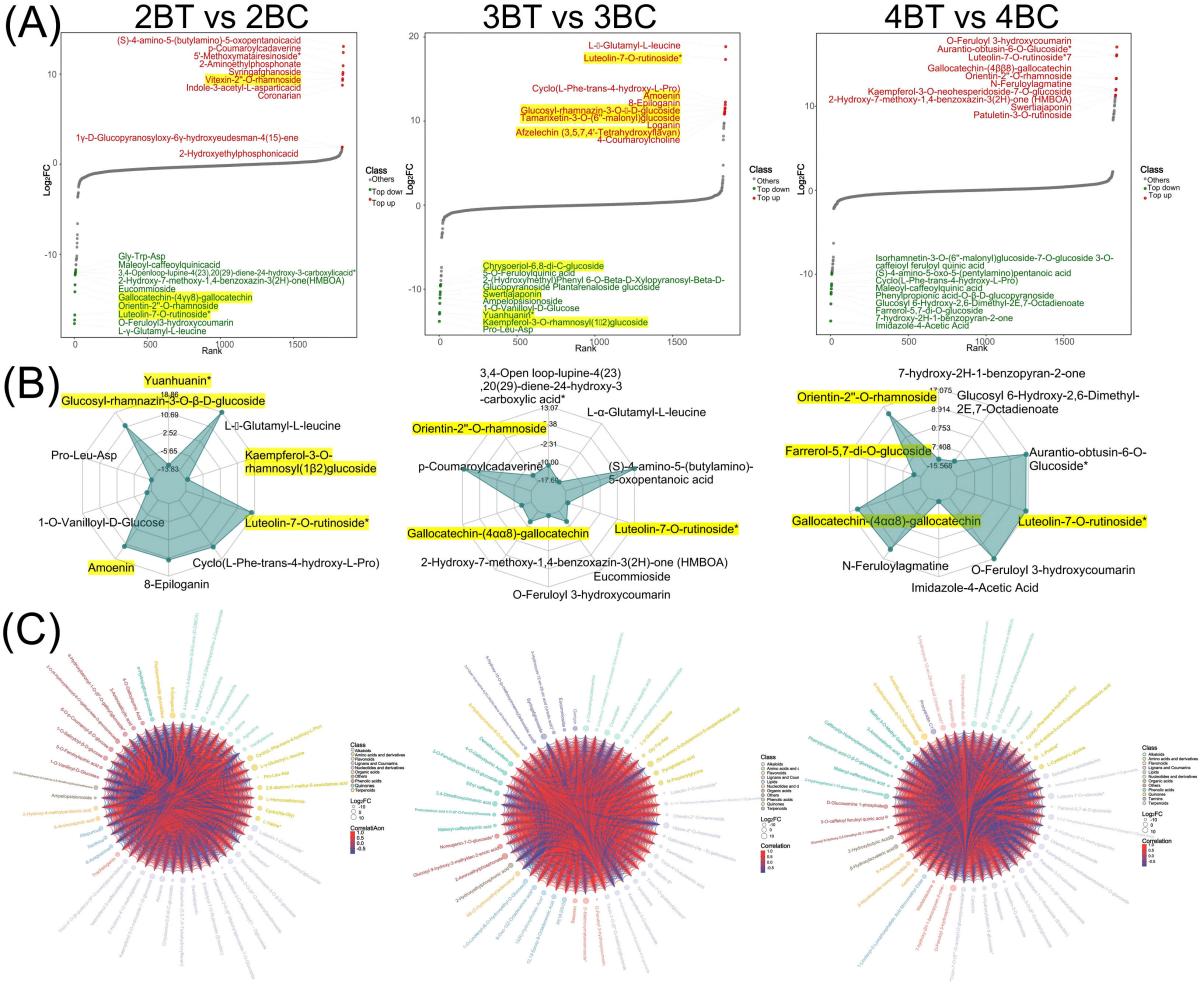
Differential metabolite analysis plot. **(A)** Map of the dynamic distribution of differences in metabolite content. Each dot represents a substance, green dots represent the top 10 down-regulated substances, red dots represent the top 10 up-regulated substances; **(B)** Radar plot of differential metabolites. The grid lines correspond to log2FC, the value of the multiplicity of differences of the differential metabolites taken logarithmically with a base of 2. The green shading consists of the log2FC connecting lines for each substance. The radar plot shows only the top 10 metabolites with the largest absolute values of log2FC; **(C)** Differential metabolite chord plot. The size of the dots represents the size of the log2FC values of the corresponding differential metabolites; the connecting lines represent the size of the Pearson correlation coefficients between the corresponding differential metabolites, with the red line representing a positive correlation and the blue line a negative correlation. When the number of differential metabolites exceeds 50, the top 50 differential metabolites with the largest VIP values are displayed.

To investigate the metabolism of blue-grained wheat seeds in response to UV-B, we performed metabolite detection based on UPLC-MS/MS on a total of 18 samples (three biological replicates per treatment) of blue-grain wheat at three different developmental stages under UV-B irradiation. The qualitative and quantitative mass spectrometry analyzes of metabolites showed that the technical reproducibility of metabolite extraction and detection in this experiment, and the stability of the instrumental status and experimental data, provided an important guarantee for the authenticity and reliability of the data and the subsequent analyzes (**Figures 2A–D**). The principal component analysis (PCA) plots showed that there was little difference between samples within the comparison group for the same period in periods 2, 3, and 4, and that period 3 (21 days after anthesis) might be the critical period for the UV-B response (**Figure 2E**). A total of 1846 metabolites were detected in this experiment, mainly flavonoids (18.42%), phenolic acids (14.52%), lipids (13%) and amino acids and their derivatives (11.43%) (**Figure 2F**). Different developmental periods of blue-grained wheat contained 138 (up:78, down:60), 160 (up:25, down:136), and 128 (up:54, down:74) differential metabolites in the pre-developmental period (2BT *vs* 2BC), mid-developmental period (3BT *vs* 3BC), and late developmental period (4BT *vs* 4BC). We screened differential metabolites based on VIP > 1 or fold change ≥ 2 and fold change ≤ 0.5, the larger the absolute value of VIP and log2FC the more significant the metabolite differences, most of the metabolites with significant differences in this experiment were flavonoids (**Figures 3A–C**; **Supplementary Figure 1**). Therefore, we deduced that flavonoids may be the key metabolites in response to UV-B irradiation, and the flavonoids will be further studied in depth.

### 3.2 Metabolomic profiling of flavonoids in blue-grained wheat in response to UV-B

**Figure 4:**
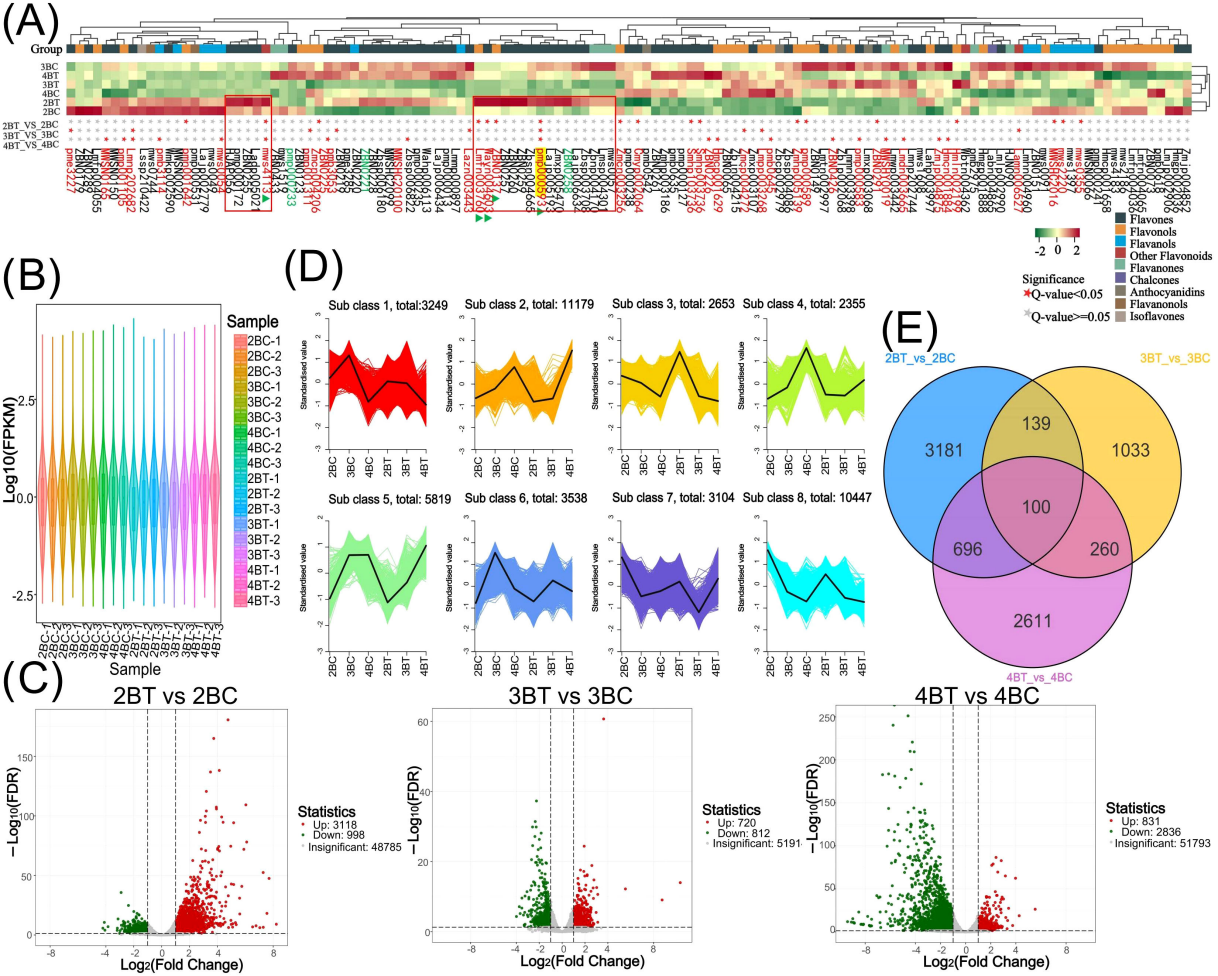
Flavonoid metabolism profiling and transcriptome overview. **(A)** Heat map of 127 flavonoids clustering. The upper part of the heat map shows the flavonoid content in different samples, and the lower part shows the significance of the flavonoids in different groups, with ★ representing significant; **(B)** Expression violin plots of genes in 18 samples of colored wheat. Different colors represent different samples and the width of each violin graph responds to the number of genes at that level of expression; **(C)** Differential gene volcano plot. Horizontal coordinates represent the fold change in gene expression and vertical coordinates represent the significance level of the differentially expressed genes. Red dots represent up-regulated differential genes, green dots represent down-regulated differential genes, and grey dots represent non-differentially expressed genes; **(D)** Differential gene Kmeans clustering plot. Horizontal coordinates indicate samples, vertical coordinates indicate normalised expression; **(E)** Venn diagram of differential genes of different subgroups. Non-overlapping areas represent differential genes specific to that differential subgroup, and overlapping areas represent differential genes common to several differential subgroups that overlap.

Further investigation was conducted based on the metabolic response of blue-grained wheat to UV-B irradiation, with the aim of identifying flavonoids as the main macromolecules in response to UV-B irradiation. A total of 340 flavonoids were identified in this experiment. Among the differential metabolites, 2BT *vs* 2BC, 3BT *vs* 3BC, and 4BT *vs* 4BC contained 72, 52, and 47 flavonoids, respectively. Eight differential flavonoids were shared among the three groups, five of which were flavones (**Supplementary Figure 2**). The number of unique differential flavonoids in the 2BT *vs* 2BC comparison was 42, in the 3BT *vs* 3BC comparison it was 22, and in the 4BT *vs* 4BC comparison it was 27. The three differential groups collectively comprised 127 flavonoids belonging to nine major categories (Flavones: 150, Flavonols: 99, Flavanones: 28, Flavanols: 19, Anthocyanidins: 13, Flavanonols:10, Other Flavonoids: 10, Chalcones: 8, Aurones: 2, Isoflavones: 1), with flavones and flavanols being the most abundant and highly expressed. The three differential groups, 2BT *vs* 2BC, 3BT *vs* 3BC, and 4BT *vs* 4BC, each contained 18, 9, and 18 significantly different flavonoids, respectively. Among these, luteolin-7-O-rutinoside (flavonoids) was significantly expressed in all three periods. In the second period, 21 flavonoids were significantly upregulated in the UV-B-irradiation group, including 15 flavanones (**Figure 4A**). The results of this study showed that the major flavonoids in blue-grained wheat in response to UV-B irradiation were flavones and flavanols.

### 3.3 Overall transcriptome profile and differential gene function analysis of blue-grained wheat in response to UV-B irradiation

**Figure 5:**
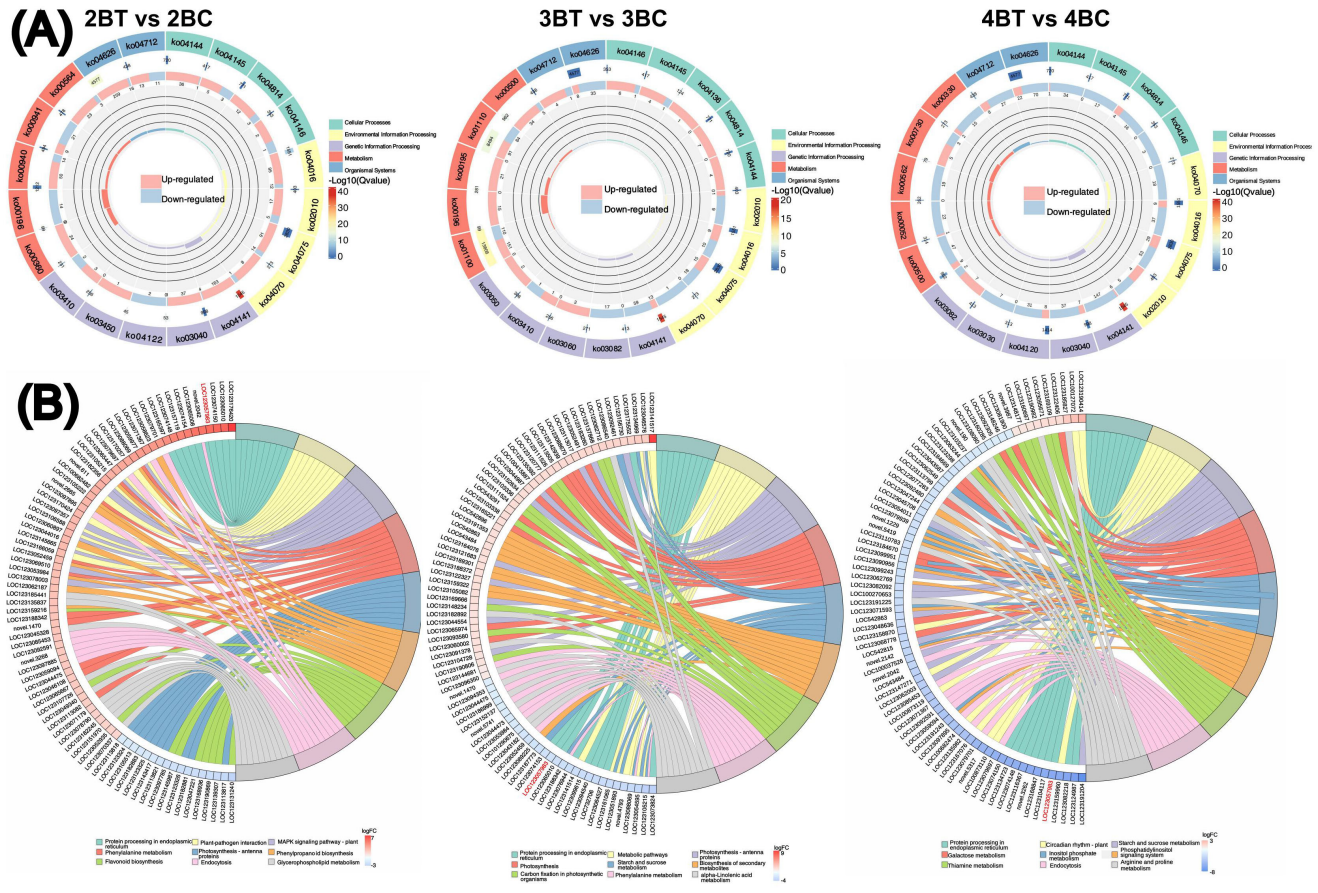
Differential gene functional annotation and enrichment analysis. **(A)** Differential gene KEGG enrichment circle diagram. From outside to inside, the first circle is the KEGG_level_1 entry, different colors represent different KEGG classifications; the second circle: the number of background genes in this classification and the q-value. the more genes the longer the bar is, the more significant the enrichment is the redder it is; the third circle: the bar of the proportion of up- and down-regulated genes, light red represents the proportion of up-regulated genes, and light blue represents the proportion of down-regulated genes; the bottom displays the specific values; Fourth circle: RichFactor value for each classification (number of foreground genes divided by number of background genes in that classification), each cell of the background auxiliary line represents 0.2; **(B)** Differential gene KEGG enrichment and chordal graphs. The graphs are divided into left and right sides: on the left side are the 10 genes with the largest |logFC| in each classification, on the right side of the graphs are the 9 pathways with the most significant enrichment, the middle line represents the correspondence between pathways and genes, the bottom right heatmap legend represents the logFC values of genes, red is for the up-regulated genes, and blue is for the down-regulated genes, and the shades of the colors indicate the size of the logFCs, and the darker the colors indicate the higher the fold of variance.

**Figure 6:**
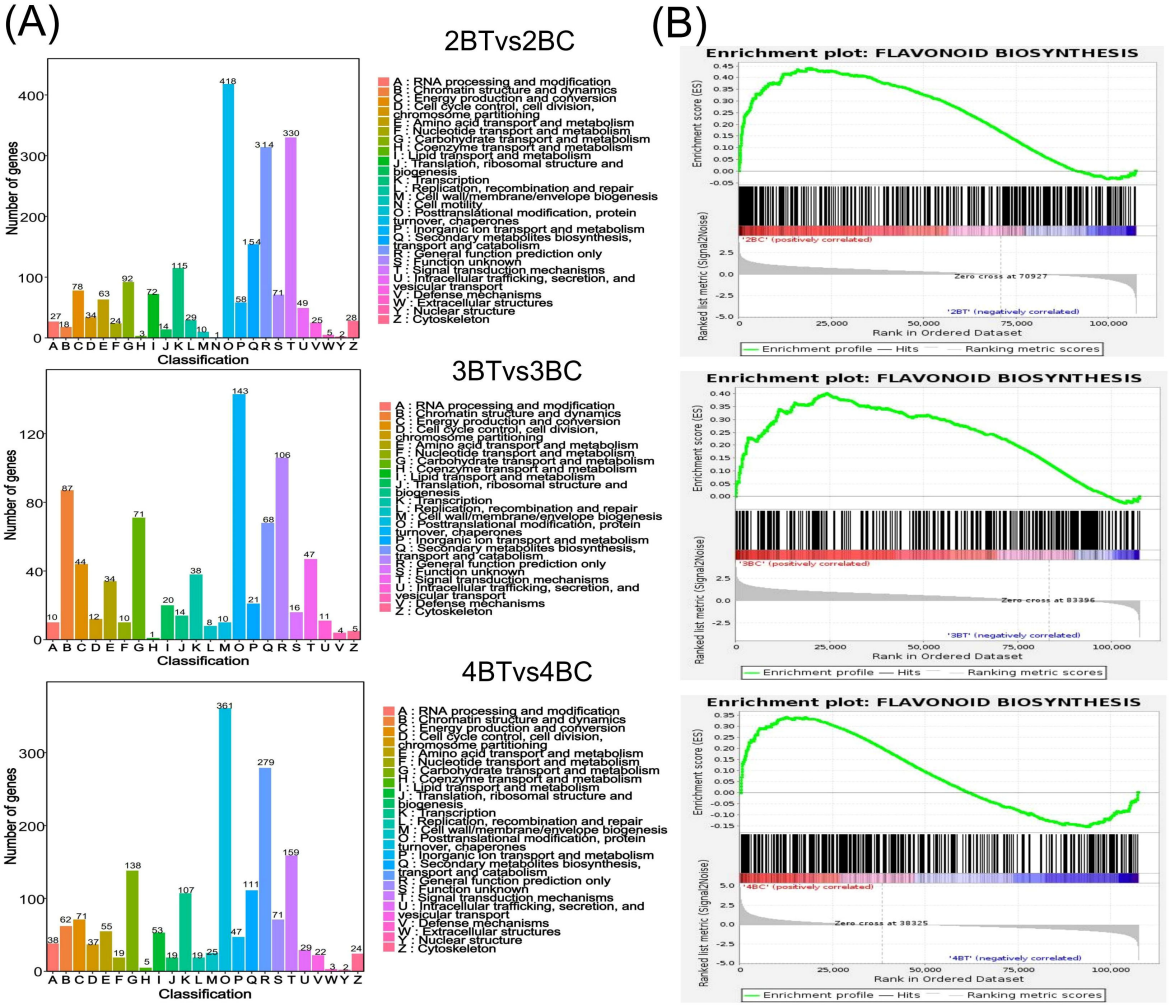
Differential gene function annotation analysis and gene set enrichment analysis (GSEA). **(A)** Bar chart of classification statistics for differential gene KOG annotations. The horizontal coordinate indicates the functional classification (Code) of the KOG ID and the vertical coordinate indicates the number of differential genes included, with different classifications indicated by different colors. The legend shows the Code plus its functional description information; **(B)** Flavonoid biosynthesis gene set enrichment analysis (GSEA) barcode plot. This graph is divided into three parts, the first part is a line graph of gene Enrichment Score, the horizontal axis is each gene under the gene, the vertical axis is the corresponding Running ES, there is a peak in the line graph, the peak is the Enrichemnt Score of the gene set, and the genes before the peak are the core genes under the gene set. The second part is the hit, with lines marking the genes located under this gene set, and the third part is the distribution of the rank values of all genes, with the Signal2Noise algorithm used by default, which corresponds to the title of the vertical axis.

A total of 18 samples of blue-grained wheat kernels from three different developmental periods under UV-B irradiation were subjected to transcriptome sequencing analysis, in order to explain how blue-grained wheat responds to UV-B irradiation at the genetic level. After filtering the raw transcriptome data, checking the sequencing error rate, and checking the distribution of GC content, a total of 238.6 Gb of Clean Data was obtained, and the Clean Data of all samples reached 8 Gb. The efficiency of the clean reads comparison to the reference genome after the quality control was more than 84%, which indicated that the results of the sequencing data could meet the needs of the subsequent analyzes. There were 42,344 differential genes in this experiment, including 3,521 novel genes and 241 MYB transcription factors related to flavonoid formation. Gene expression was higher in the late stage of blue-grained wheat than in the early stage (**Figure 4B**), and UV-B-irradiation blue-grained wheat had more up-regulated material in the early stage and more down-regulated material in the late stage (**Figure 4C**), indicating that blue-grained wheat mainly up-regulated genes in the early stage in response to UV-B irradiation. Compared with the control group, 2653 genes were highly expressed in the pre-developmental stage and 16998 genes were highly expressed in the post-developmental stage of the kernel in the UV-B-irradiation blue-grained wheat at different periods (**Figure 4D**), and the highest number of pre-developmental-specific differential genes was as high as 3181 (**Figure 4E**), which reconfirmed that the blue-grained wheat mainly expresses genes in the pre-developmental stage in response to the UV-B irradiation. The three period comparison groups of differential genes were analyzed for KEGG functional annotation and enrichment, and only 2BT *vs* 2BC contained the Ko00940 and Ko00941 pathways associated with flavonoid formation, with a large proportion of genes up-regulated in the first two periods in general by UV-B irradiation, however, a high number of genes down-regulated in the last period by UV-B irradiation (**Figure 5A**). In the differential gene GO function annotation and analysis, there was a shift from more up-regulated genes to more down-regulated genes with the developmental period under UV-B treatment (**Supplementary Figure 3A**). Differential gene KEGG and GO enrichment analyzes showed that the common enrichment pathway for KEGG in the three periods of treatment groups were Protein processing in endoplasmic reticulum, with a common differential gene *LOC123057983* (**Figure 5B**), and the common enrichment pathway for GO in the three periods of treatment groups was response to hydrogen peroxide, protein complex oligomerization, shared differential genes *LOC123065010* and *LOC123057983* (**Supplementary Figure 3B**). Clustering KOG analysis of homologous proteins of differentially expressed genes in color wheat showed that O: posttranslational modification, protein turnover, chaperones and R: secondary metabolites biosynthesis, transport and catabolism had the highest number of genes in the UV-B-treated comparison group at different times, and the number of T: Signal transduction mechanisms relevant to this study is higher in the predevelopmental genes (**Figure 6A**). We performed KEGG and GO GSEA based on genes that were not significantly differentially expressed but biologically important in addition to the significantly differentially expressed genes, 2BT *vs* 2BC, 3BT *vs* 3BC and 4BT *vs* 4BC screened for 136 pathways in KEGG GSEA with a total of 107,474 genes, the highest enrichment scores were for photosynthesis - antenna proteins, phenylpropanoid biosynthesis, protein processing in endoplasmic reticulum, and 99 gene sets were highly expressed under UV-B treatment in the late developmental stages of blue-grained wheat. 2BT *vs* 2BC, 3BT *vs* 3BC and 4BT *vs* 4BC all had 28 gene sets in GO GSEA, and the highest enrichment scores were for structural molecule activity, antioxidant activity, and protein folding chaperone, respectively.

### 3.4 Expression analysis of flavonoid biosynthesis-related genes in blue-grained wheat in response to UV-B and validation by quantitative real-time polymerase chain reaction

**Figure 7:**
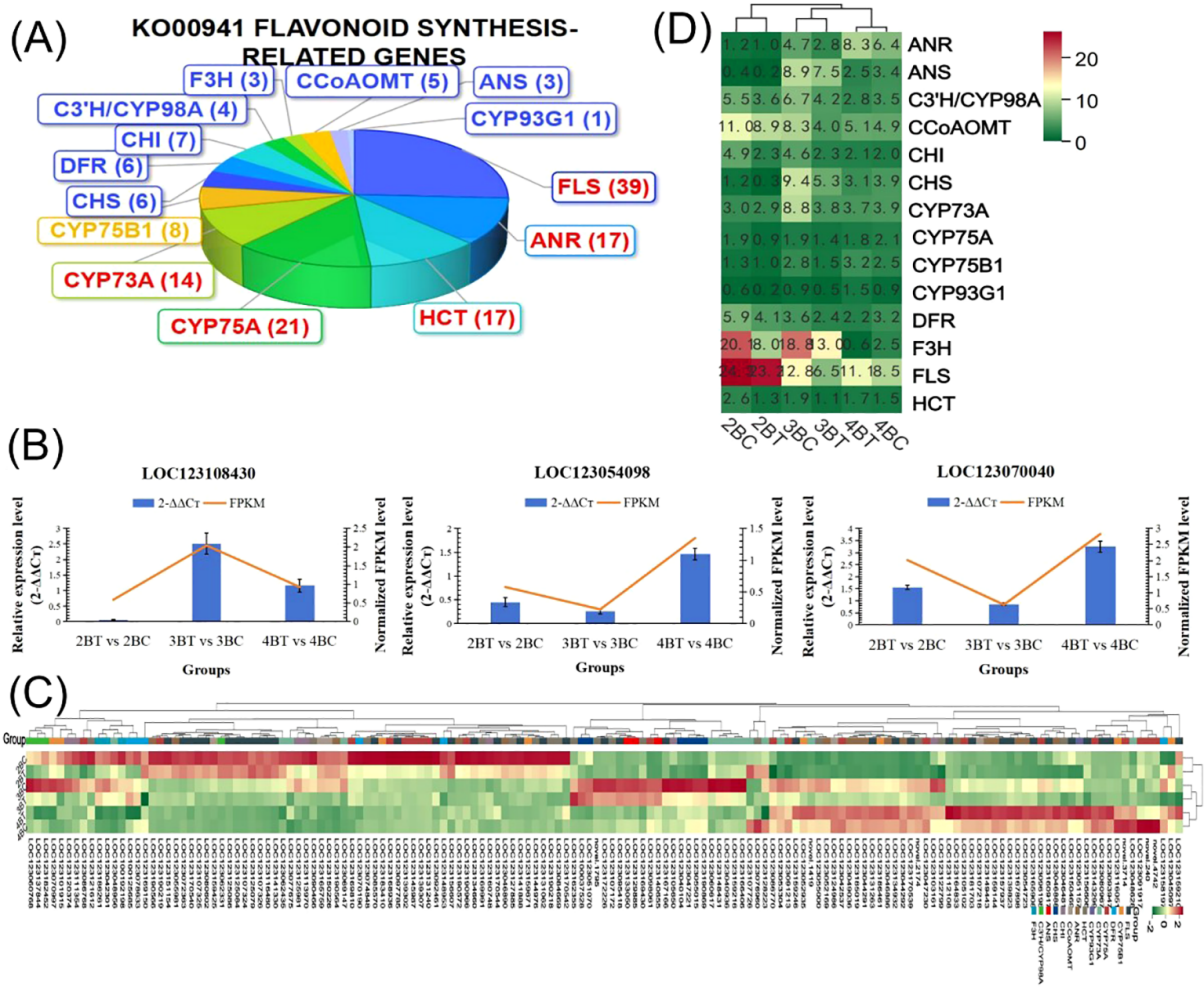
Genes related to flavonoid biosynthesis pathways. **(A)** Pie chart of 151 differential gene classifications in the Ko00941 flavonoid pathway; **(B)** Quantitative Real-time PCR Validation; **(C)** Heatmap of the expression of 151 flavonoid synthesis-related genes, with high expression in red; **(D)** Heatmap of 151 flavonoid synthesis-related gene classifications, 14 classifications on the left, high expression in red.

Metabolomic assays identified predominantly flavonoids in blue-grained wheat in response to UV-B irradiation, and thus the flavonoid synthesis pathway was the focus of this study. Our analysis of the flavonoid biosynthesis gene set, which was not significantly differentially expressed, revealed that it contained 362 genes, 96 of which were enriched core genes, and that UV-B-treated wheat flavonoids were down-regulated (**Figure 6B**). In addition, the genes with significant differential expression were annotated into the Ko00941 flavonoid pathway with 151 genes, and 151 genes were annotated by Pfam into PF02431.18: Chalcone-flavanone isomerase (7), PF00195.22:Chalcone and stilbene synthases, N-terminal domain (6). 151 flavonoid biosynthesis-related genes were classified into 14 classes of genes, including flavonol synthase (*FLS*), anthocyanidin reductase (*ANR*), shikimate O- hydroxycinnamoyltransferase (*HCT*), flavonoid 3’,5’-hydroxylase (*CYP75A*), and trans-cinnamate 4-monooxygenase (*CYP73A*) genes were more in number (**Figure 7A**), of which only gene *LOC123107726* is a *FAR1* transcription factor. qRT-PCR was performed to validate the randomly selected differentially expressed genes, and each reaction was repeated three times, and 2^-ΔΔCT^ was taken to analyze the normalized expression of each sample, and the results showed that the expression patterns detected by qRT-PCR correlated well with the sequencing results, indicating the reliability of the transcriptome sequencing results (**Figure 7B**). The 151 genes were subjected to gene expression profiling, and it was found that the genes treated with UV-B in period 4 were significantly higher than the control expression, among which the expression of *F3H* (naringenin 3-dioxygenase [EC:1.14.11.9]) and *FLS* (flavonol synthase [EC:1.14.20.6]) was higher, among which the expression was decreased in both period 2 and 3 after UV-B treatment, and the expression of some of the genes was increased in period 4 (**Figures 7C, D**).

### 3.5 Combined transcriptomic and metabolomic analysis of flavonoid biosynthesis in blue-grained wheat under UV-B irradiation

**Figure 8:**
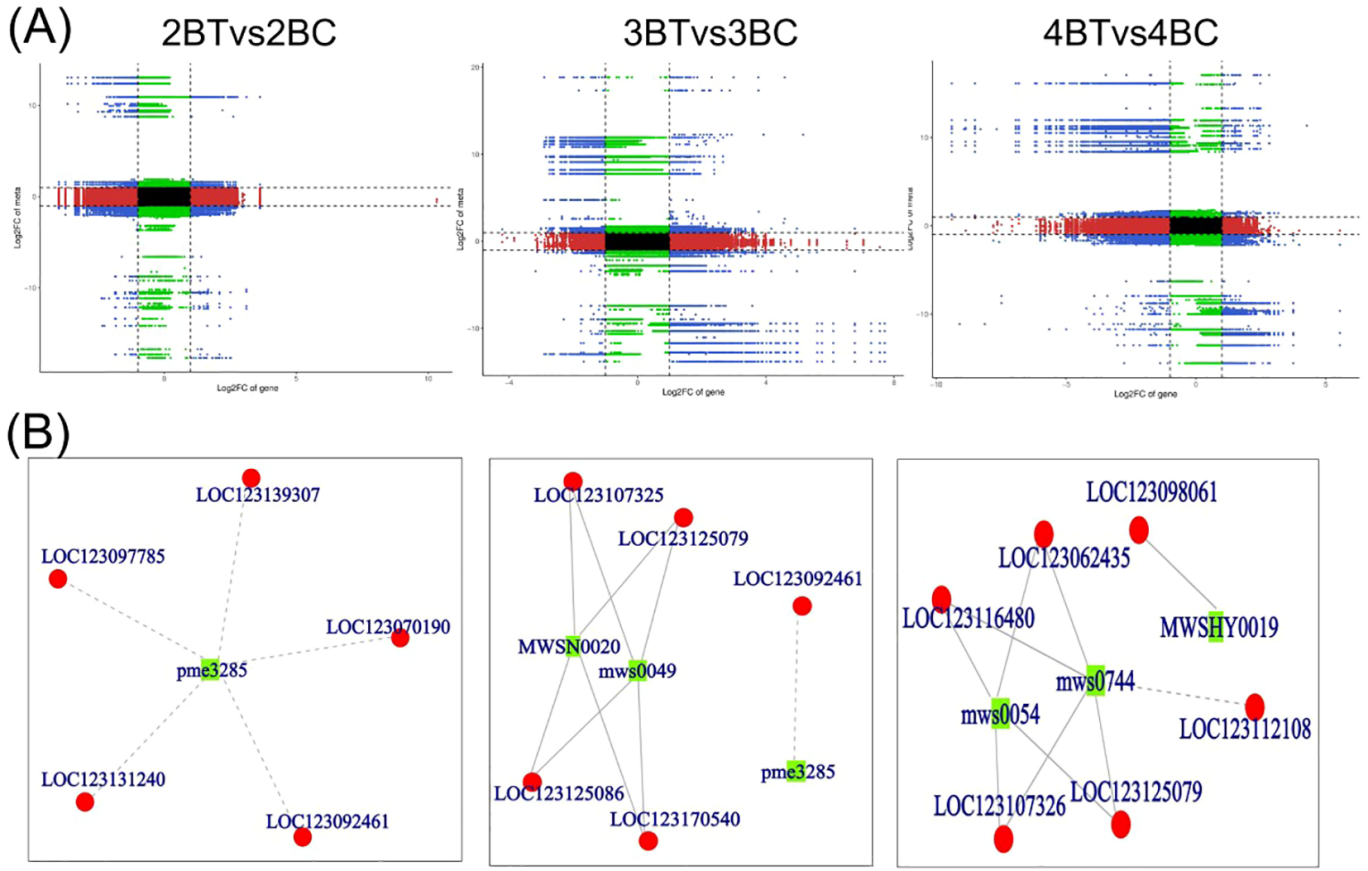
Transcriptome and metabolome correlation analysis of flavonoid synthesis pathways in blue-grained wheat in response to UV-B. **(A)** Nine-quadrant plot for gene and metabolite correlation analysis. Nine-quadrant plots showing the multiplicity of differences for substances with Pearson correlation coefficients greater than 0.80 and pvalues less than 0.05 in each subgroup of differences, using black dashed lines, from left to right and top to bottom, in quadrants 1-9, with the horizontal coordinates indicating the log2FC for the genes and the vertical coordinates indicating the log2FC for the metabolites; **(B)** Network diagram of gene and metabolite correlations. Metabolites are marked with green squares and genes are marked with red circles in the figure. Solid lines represent positive correlations and dashed lines represent negative correlations.

**Figure 9:**
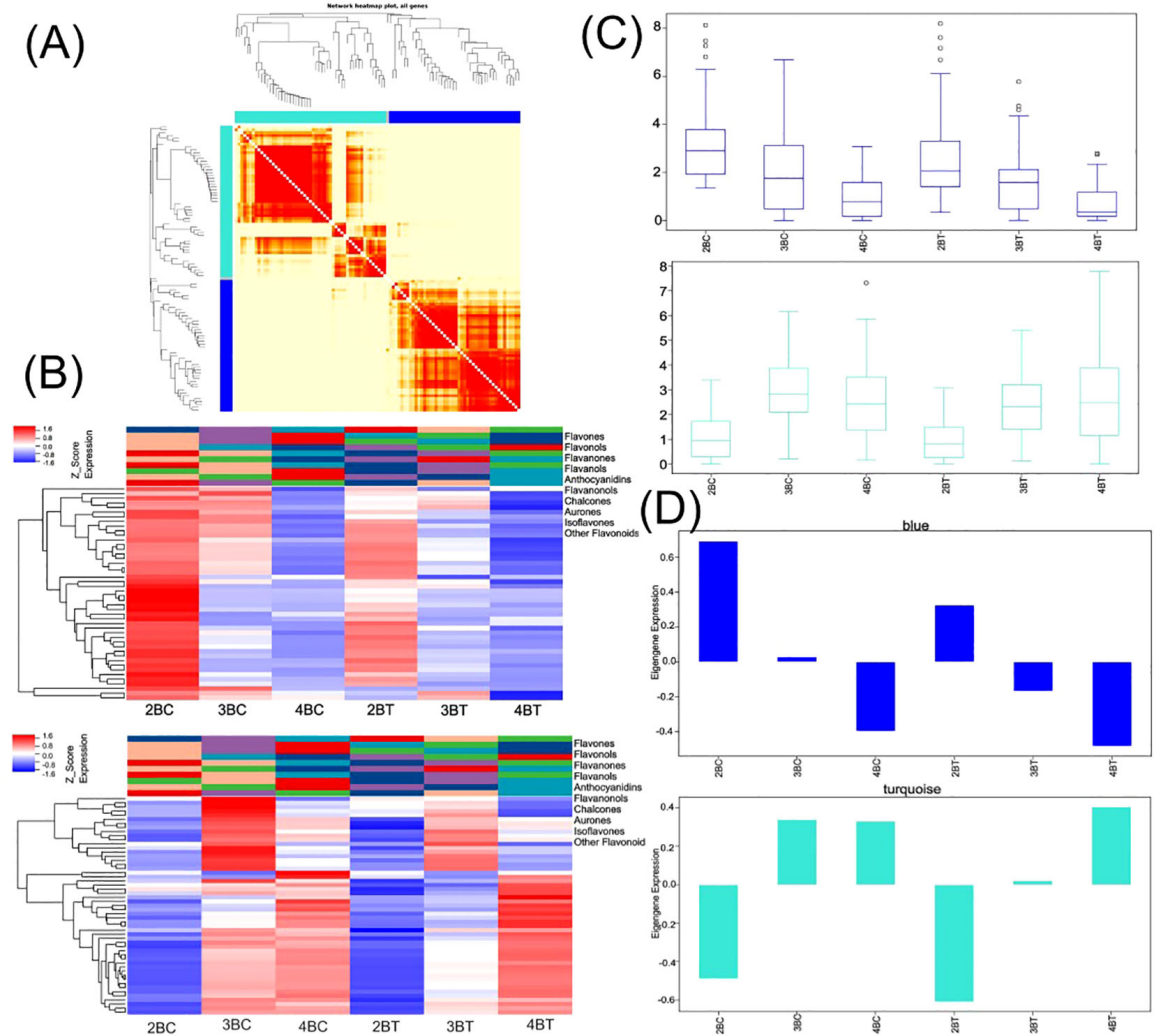
Multi-omics combined with WGCNA analysis of flavonoid biosynthesis in blue-grained wheat under UV-B. **(A)** Heat map of modular genes based on WGCNA analysis; **(B)** Blue module and turquoise module gene heatmap; **(C)** Blue module and turquoise module box line diagrams; **(D)** Blue module and turquoise module feature gene bar graphs.

**Figure 10:**
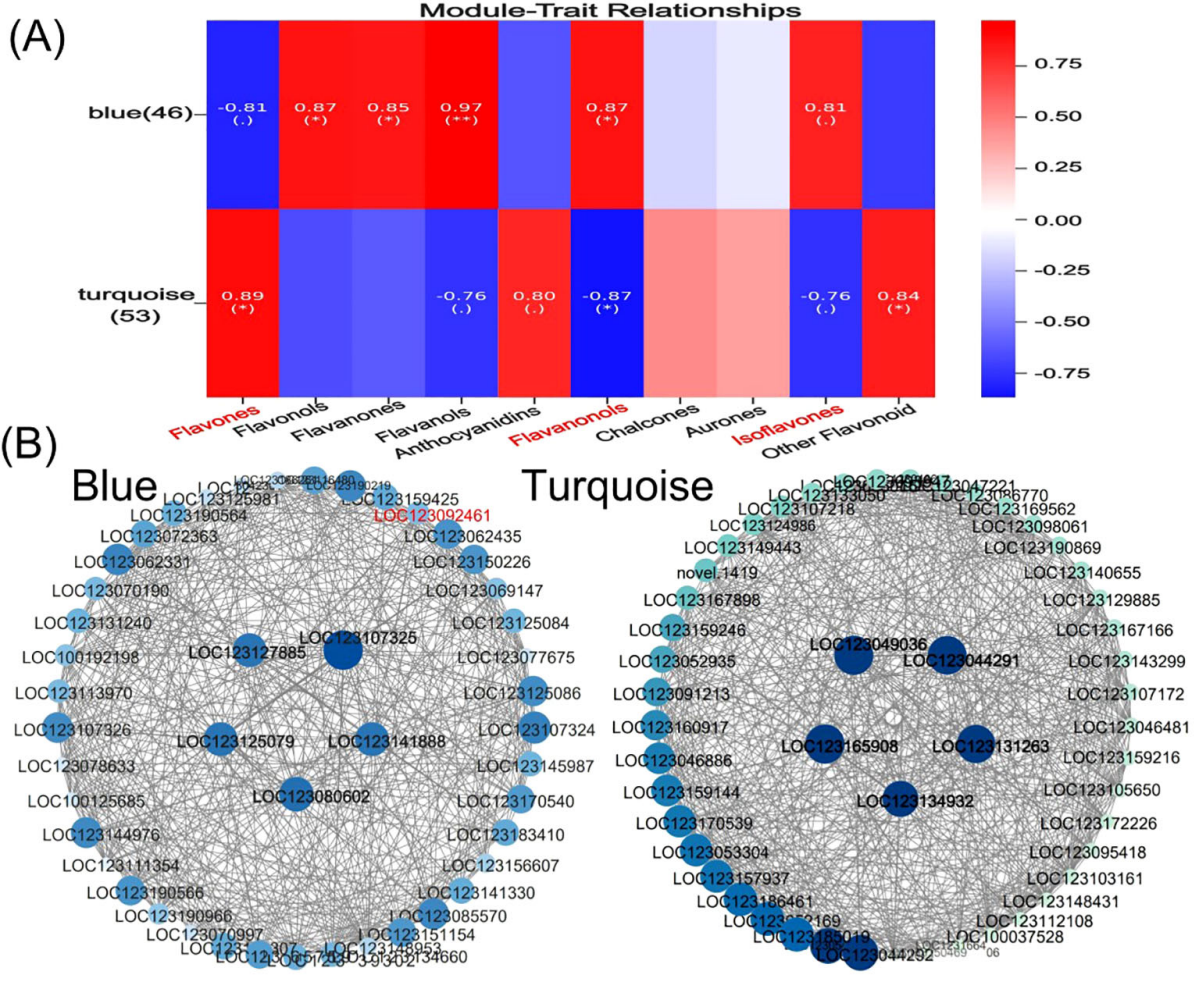
Core gene mining for flavonoid biosynthesis in blue-grained wheat under UV-B irradiation using WGCNA. **(A)** Heatmap of module and flavonoid correlation analysis. Red represents positive correlation, blue represents negative correlation; **(B)** Network diagram of core gene correlation for Blue module and turquoise module. Circle size and color shades represent correlation strength.

Flavonoid synthesis-related metabolites increased with developmental period in blue-grained wheat in response to UV-B in transcriptome and metabolome correlation analyzes of the flavonoid synthesis pathway (Flavonoid Synthesis Pathway 941). In the gene and metabolite differential expression pattern correlation analysis (consistent and opposite gene and metabolite expression patterns), 2457/116/61, 1810/119/31, and 2805/108/38 genes/metabolites/flavonoids were found in the predevelopmental, mid-developmental, and post-developmental stages of blue-grained wheat, respectively, in the presence of UV-B irradiation, which means that flavonoid biosynthesis of blue-grained wheat in response to UV-B irradiation was mainly in the pre-developmental stage (**Figure 8A**). The results of the correlation network diagram of differential genes and differential metabolites in the flavonoid synthesis pathway showed that metabolites were negatively correlated with genes in the pre-developmental stage of wheat grain, while metabolites and genes were positively correlated in the mid- and late-developmental stages (**Figure 8B**). Based on the joint transcriptome and metabolome analysis, we analyzed the flavonoid biosynthesis of blue-grained wheat under UV-B irradiation by using the flavonoid content in the metabolome as the basis of the trait for mining WGCNA. The flavonoid synthesis pathway was filtered for low-expressed genes for WGCNA data analysis, which was divided into turquoise, blue, grey totaling 3 modules with 53, 46, and 1 genes (**Figure 9A**), respectively, and we followed up with in-depth analyzes of the modules turquoise and blue. The turquoise module showed a positive feedback in gene expression by UV-B treatment at period 4, the blue module showed a negative feedback in gene expression by UV-B treatment (**Figure 9B**), and both modules showed an overall decrease in expression with developmental time (**Figure 9C**), and the turquoise module genes were highly expressed by UV-B treatment at period 4 (**Figure 9D**). Both modules were strongly correlated with flavones, flavanonols and isoflavones with opposite correlations (**Figure 10A**). We constructed a correlation network map of the modules (**Figure 10B**), and each module was screened for five core genes each, in which the transcriptome and metabolome correlation analyzes of the flavonoid synthesis pathway, *LOC123092461*, were in the blue module (the core genes of the blue module were *LOC123107325*, *LOC123080602*, *LOC123125079*, *LOC123141888* and *LOC123127885*, and turquoise module core genes are *LOC123165908*, *LOC123049036*, *LOC123044291*, *LOC123131263* and *LOC123134932*).

### 3.6 Analysis of flavonoids synthesis pathway in blue-grained wheat under UV-B irradiation

**Figure 11:**
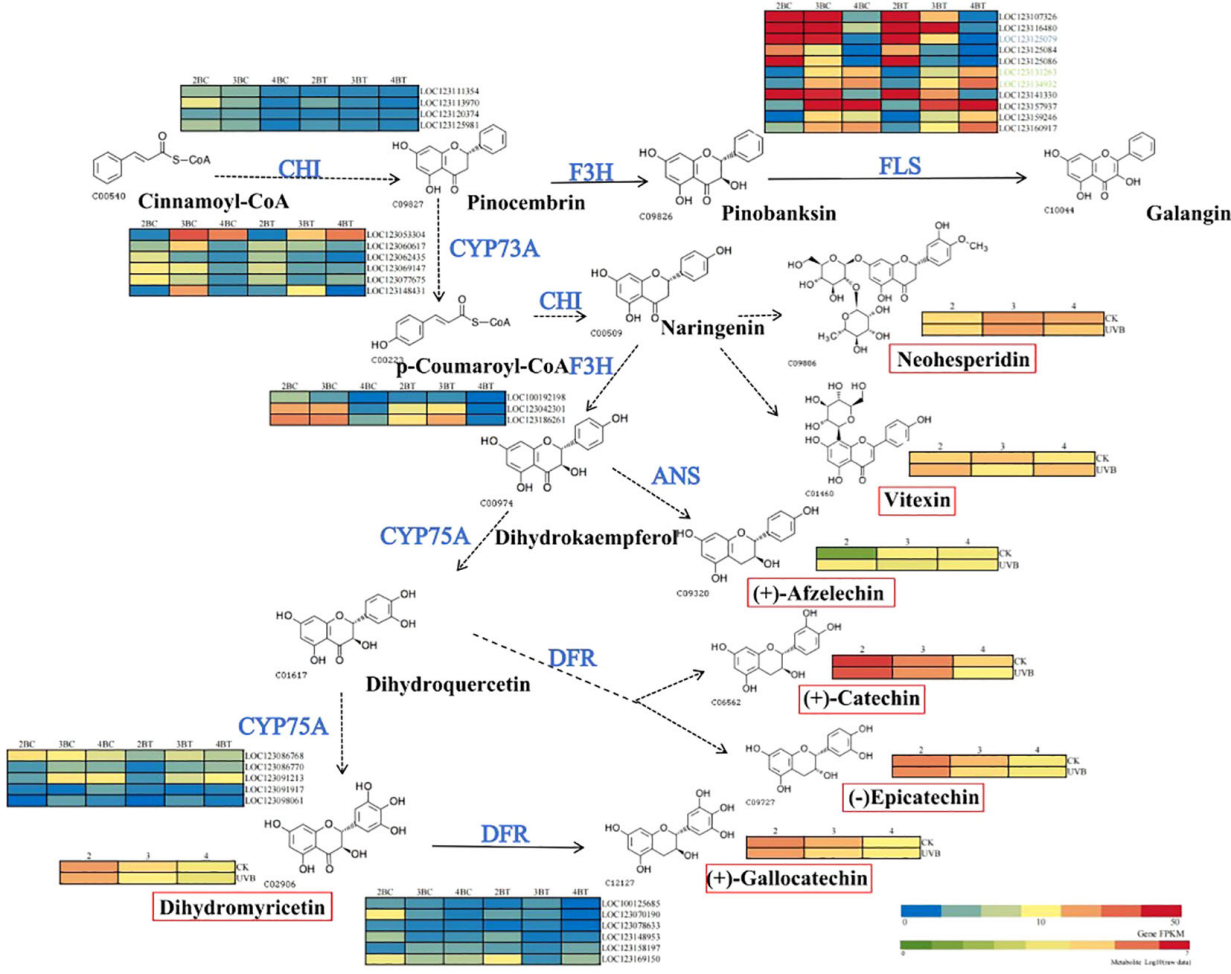
Mechanism of flavonoid synthesis in blue-grained wheat under UV-B. The figure is based on the flavonoid synthesis pathway, labelled as constructed by key nodes of metabolites and genes in colored wheat under UV-B irradiation, with key enzyme cleavage sites in blue font, and the red color of the gene heatmap of the enzyme cleavage sites representing higher gene expression, and the metabolites in the flavonoid synthesis pathway in black, with the degree of redness representing the higher metabolite content. FLS, flavonol synthase; CYP75A, flavonoid 3’, 5’-hydroxylase; CYP73A, trans-cinnamate 4-monooxygenase; CHI, chalcone isomerase; F3H, flavonoid3’-hydroxylase; ANS, Anthocyanidin synthase; DFR, Dihydroflavonol reductase.

In the flavonoid biosynthetic pathway, there is a greater number of differentially expressed genes in the second and fourth periods as metabolites increase with developmental time differences. In the context of UV-B treatment, there are seven different metabolites and seven flavonoid biosynthesis-related genes. Among these, dihydromyricetin is associated with anthocyanin biosynthesis. The concentrations of (+)-catechin, (-)-epicatechin, (+)-gallocatechin, and dihydromyricetin decrease with developmental time, whereas those of vitexin accumulate in the second and fourth periods under UV-B treatment. In the pathway of flavonoid biosynthesis-related enzymes, the majority of genes in *CHI* and *DFR* are downregulated under UV-B treatment in comparison to the control group. FLS is highly expressed throughout the flavonoid biosynthetic pathway. Furthermore, *LOC123125079*, *LOC123131263*, and *LOC123134932* are core genes selected through WGCNA. The core gene *LOC123125079* exhibited a pronounced decline in expression during the third period under UV-B treatment (**Figure 11**). In consideration of the entire mechanism, UV-B exerts a significant influence on the accumulation of seven flavonoid metabolites in blue-grained wheat, with the most pronounced impact observed during the early stages. *FLS* is a key factor influencing the accumulation of flavonoids in blue-grained wheat, with the core gene *LOC123125079* potentially playing a pivotal role in the response of blue-grained wheat to UV-B.

## 4 Discussion

The extensive cultivation and consumption of wheat has led to it becoming the main source of heat and protein. Color wheat contains a greater quantity of anthocyanins and exhibits stronger antioxidant activity than common wheat (Hong et al., 2024). With environmental changes, there is an increasing interest in plant response to UV-B irradiation, which affects plant cell damage, flavonoid accumulation and photosynthesis (Jenkins, 2009; Kataria et al., 2014; Nawkar et al., 2013). Generally speaking plant exposure to acute UV-B environments produces more severe effects than chronic UV-B environments (Wilson and Ruban, 2019) and there are different responses to UV-B acclimation in plants at the epigenetic and transcriptional levels (Wang et al., 2021). Most of the current studies on UV-B effects on plants are at the level of physiological and morphological changes. However, the material in this experiment was planted in Kunming, Yunnan Province, where the plateau has strong UV-B irradiation. We aimed to dissect the metabolome and transcriptome to find out the reasons for the dynamic changes of flavonoid accumulation and related metabolic gene expression in blue-grained wheat under UV-B irradiation.

UV-B irradiation is a potential pathway to improve antioxidant nutrients and phytochemicals (Jiao et al., 2016), affecting plant phenolics and flavonoids and thus indirectly influencing quality formation. Simple UV-B supplementation strategies can affect the concentration of plant flavonoids, phenolics, anthocyanins and ascorbic acid, as well as their antioxidant capacity, thereby altering their flavor profiles and nutritional properties and making them more attractive for processing into functional foods (Lin et al., 2021; Gai et al., 2022; Wittayathanarattana et al., 2022, 2022; Lin et al., 2021). Flavonoids were the main metabolites in response to UV-B irradiation as in most plants in this study, but the number of flavonoids decreased with the difference in developmental time, among which luteolin-7-O-rutinoside (Flavanols) was significant in the comparison groups of the three time periods, and may be the specific flavonoids in response to UV-B irradiation in blue-grained wheat. The second developmental stage exhibited the greatest upregulation of flavonols in response to UV-B irradiation, indicating that blue-grained wheat has the greatest impact on flavonol biosynthesis after UV-B irradiation in the early stage. Immediately following UV-B irradiation, the plant responds with the upregulation of 15 types of flavanones. In conclusion, in response to UV-B environmental stimuli, blue-grained wheat, like other plants, produces flavonol substances as protective agents, primarily flavanols and flavanones. The most significant response occurs in the early stage before environmental stimuli, and the response of flavonol substances subsequently declines as the plant adapts to the environmental stimuli over time.

Transcriptomic responses triggered by UV-B irradiation have been extensively studied in plants, with different UV irradiation intensities promoting different levels of expression of genes for secondary metabolite biosynthesis (Lee et al., 2022). A multitude of investigations have demonstrated that UV stimulation upregulates enzymes associated with flavonoid synthesis, including *PAL*, chalcone synthase (*CHS*), and chalcone isomerase (*CHI*), which collectively promote the accumulation of phenolic compounds and flavonoids as secondary metabolites for UV-B protection (Frohnmeyer and Staiger, 2003; Fuglevand et al., 1996). Many studies have demonstrated delayed gene expression after UV-B irradiation (Clayton et al., 2018). UV-B irradiation resulted in changes in the chalcone synthase-related isoflavone biosynthesis genes *GmCHS6*, *GmCHS7*, and *GmCHS8* in soybean seeds (Lim et al., 2020), and *CsHY5*-mediated activation of *MYB12* in tea and binding to the promoter of flavonoid biosynthesis genes, leading to changes in flavonoids (flavanols) and the formation of bitter and astringent flavors (Lin et al., 2021; Zhao et al., 2021). The results of our experiment are consistent with those of previous studies, indicating that the UV-B response in blue-grained wheat is primarily associated with flavonoids, with a particular focus on genes such as *FLS*, *ANR*, *HCT*, *CYP75A*, and *CYP73A*. Notably, the gene *LOC123107726* is a FAR1 transcription factor, which may be related to crop heterogeneity.

During the defensive strategies employed by plants to counteract the effects of UV irradiation, the protective mechanism of UV-B irradiation is mediated by the upregulation of genes such as GPP, HMG-CoA reductase, DXS, DAHP, and PAL, resulting in an enhanced biosynthesis of phenylalanine, carotenoids, lutein, and anthocyanins to mitigate UV damage (Ghasemi et al., 2019; Casati and Walbot, 2003). In addition, DNA methylation is also a way to adapt to UV-B irradiation (Zhao et al., 2024). In conclusion, moderate UV-B promotes the accumulation of flavonoids in plants and severe UV-B irradiation adversely affects the photosynthetic capacity of plants (Kataria and Guruprasad, 2015). UV-B supplementation strategies are often utilized in agriculture to promote nutrient accumulation in crops, e.g., the *PAL* in groundnut and cucumber was up-regulated to the highest expression level after UV-B irradiation (Qian et al., 2019). That is, genes related to the flavonoid biosynthesis pathway may be expressed during or after UV-B irradiation and activate flavonoid biosynthesis. Because UV irradiation is easy to regulate and does not induce chemical contaminants during treatment, research on UV-B irradiation of plants at specific thresholds to increase the production of plant secondary metabolites and enhance the content of bioactive compounds is increasing (Diago et al., 2012; Jiao et al., 2015; Lee et al., 2021). In this study, wheat treated with UV-B exhibited up-regulation of more substances in the early stage and down-regulation of more substances in the later stage, with significant enrichment of pathways related to flavonoid formation observed only in 2BT *vs* 2BC, indicating that the primary regulation of flavonoid biosynthesis in blue-grained wheat occurs in the early stage. The expression levels of *F3H* and *FLS* were found to be high in this study. Following UV-B treatment in the second and third periods, there was a decrease in expression levels for some genes, while in the fourth period, there was an increase in expression levels for other genes. The flavonoid biosynthesis pathway is characterized by the high expression of *FLS*. The core gene *LOC123125079*, which plays a pivotal role in this pathway, exhibited a significant decline in expression levels following UV-B treatment in the third period.

In summary, blue-grained wheat exhibits a robust response to UV-B environmental stimuli, defending against UV-B damage to the plant itself. This response is most pronounced in the early stages. Once the plant has adapted to the environmental stimulus, the gene response may not be as pronounced, but the accumulation of plant metabolites does require some time. Consequently, harvesting plants immediately after UV irradiation may not be an optimal approach, as the biosynthesis of biologically active compounds may not be complete (Wittayathanarattana et al., 2022). The results of this experiment demonstrate the efficacy of a strategy for supplementing UV-B irradiation to enhance the accumulation of biologically active compounds, such as flavonoids.

## 5 Conclusion

UV-B irradiation represents a potential pathway for the enhancement of antioxidant nutrients and phytochemicals, with a particular impact on the accumulation of plant flavonoid metabolites. This, in turn, exerts an indirect influence on the formation of quality. The distinctive nutritional attributes of blue-grained wheat are intimately linked to the flavonoids present in its pericarp layer, rendering the investigation of flavonoid accumulation in blue-grained wheat under UV-B treatment a crucial endeavor. In this study, a total of 1846 metabolites were identified in blue-grained wheat grains under UV-B treatment, including 340 flavonoids. The number of flavonoid metabolites decreased with the progression of development under UV-B treatment. A total of 127 differentially expressed flavonoid substances from nine subclasses were identified among the three groups. The most abundant and highly expressed classes were flavones and flavanols. Luteolin-7-O-rutinoside (flavanols) exhibited significant expression in all three periods. The expression of flavonoids was found to be significantly upregulated in the second period under UV-B treatment. A total of 42,344 differentially expressed genes were identified from transcriptomic analysis, including 3,521 novel genes. A total of 241 *MYB* transcription factors related to flavonoid formation and 151 genes in the Ko00941 flavonoid pathway, divided into 14 gene families, were identified. The pathway exhibited a higher number of *F3H* and *FLS* genes. The expression levels of *F3H* and *FLS* decreased in the second and third periods following UV-B irradiation, while some genes exhibited increased expression in the fourth period. In the joint analysis of the two groups, *F3H* and *FLS* enzymes were identified as key enzyme cleavage sites in our study. *LOC123125079* in *FLS* was found to play a crucial role in the response of blue-grained wheat to UV-B irradiation. The results provide a theoretical and experimentally foundation for studies on supplementing UV-B irradiation before harvest to increase the content of bioactive compounds, which is of great significance for exploring the unique nutritional characteristics of colored wheat.

## Data Availability

The original contributions presented in the study are publicly available. This data can be found here: National Center for Biotechnology Information (NCBI) SRA database under bioproject accession number PRJNA1114371.
