# Supplementary material for: UV-B induced flavonoid accumulation and related gene expression in blue- grained wheat at different periods of time
Source: Front Plant Sci. 2024 Dec 16;15:1520543. doi: 10.3389/fpls.2024.1520543 (PMC11684391; doi:10.3389/fpls.2024.1520543)
Supplement: Supplementary file 1 [file DataSheet1.zip › supplementary figure/supplementary figure.docx]

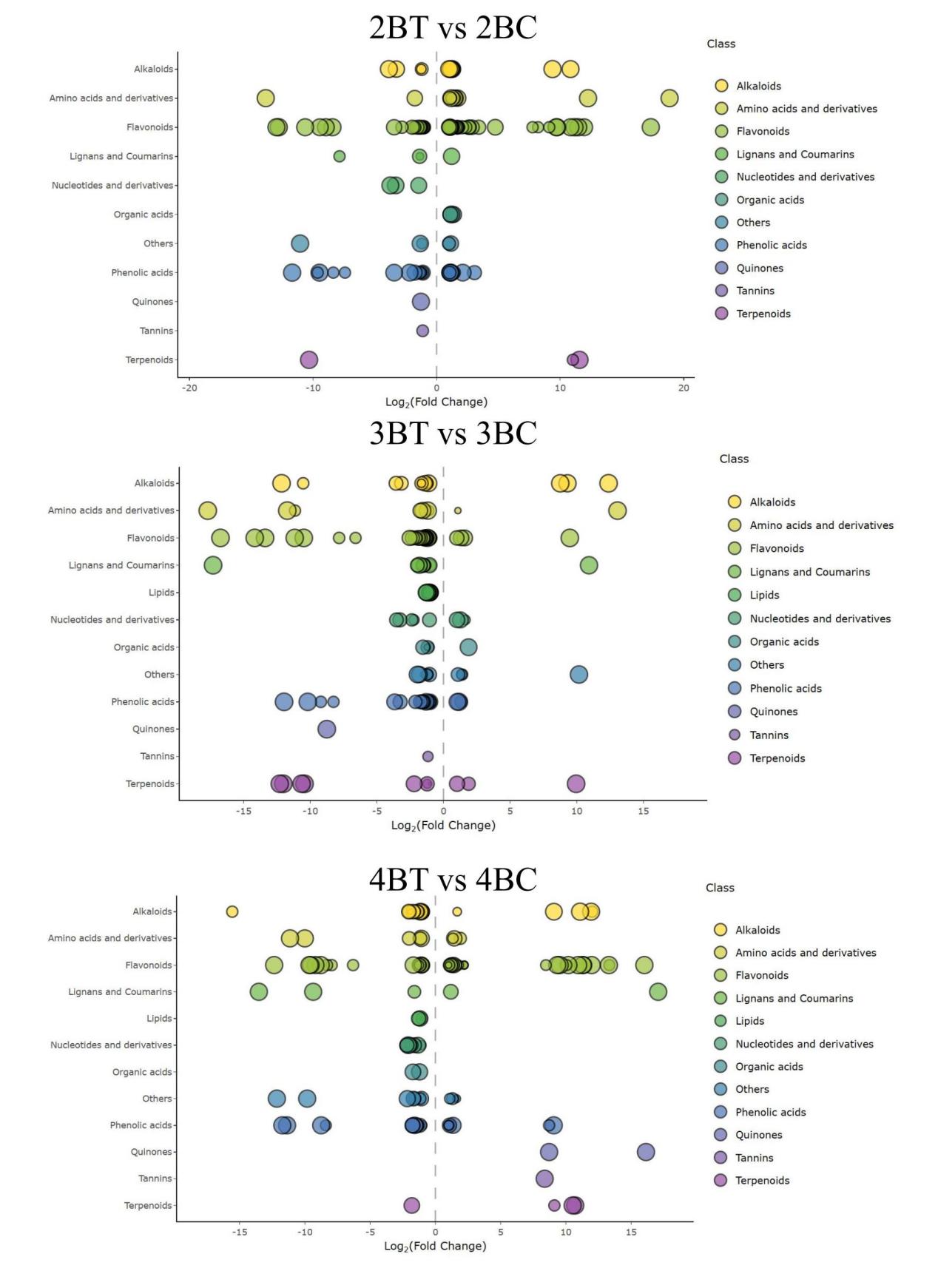


**Fig.S1.**
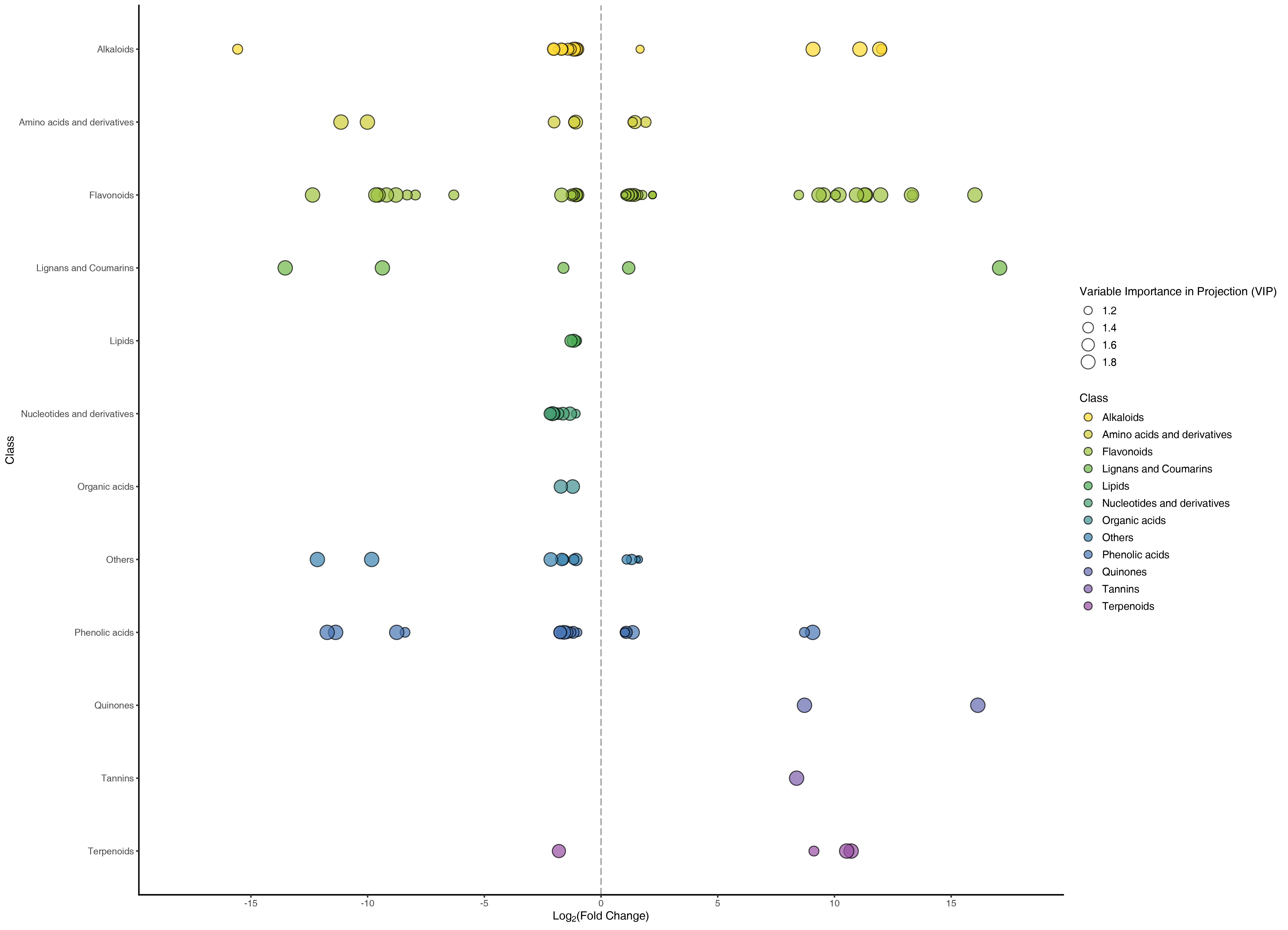
 Differential Metabolite Scatter Plot


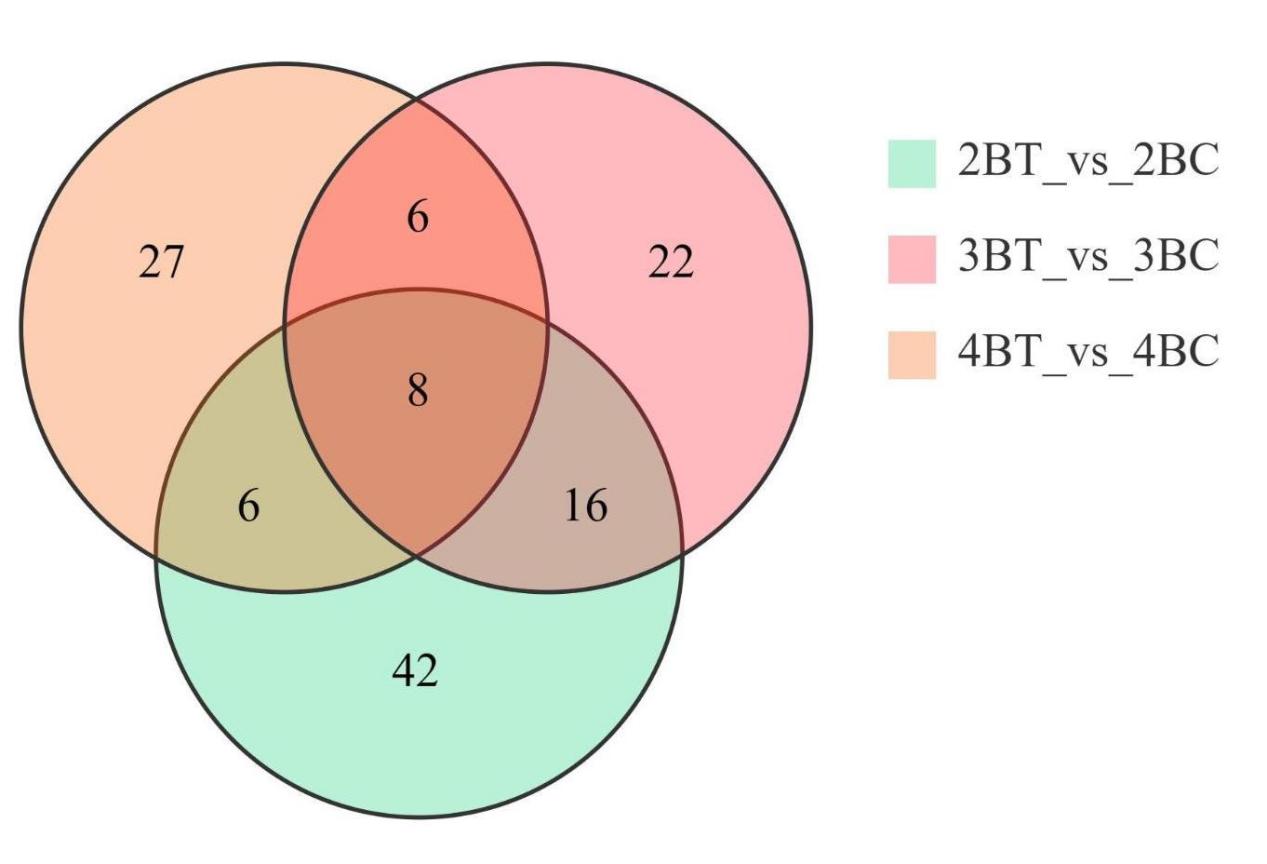


**Fig.S2.**
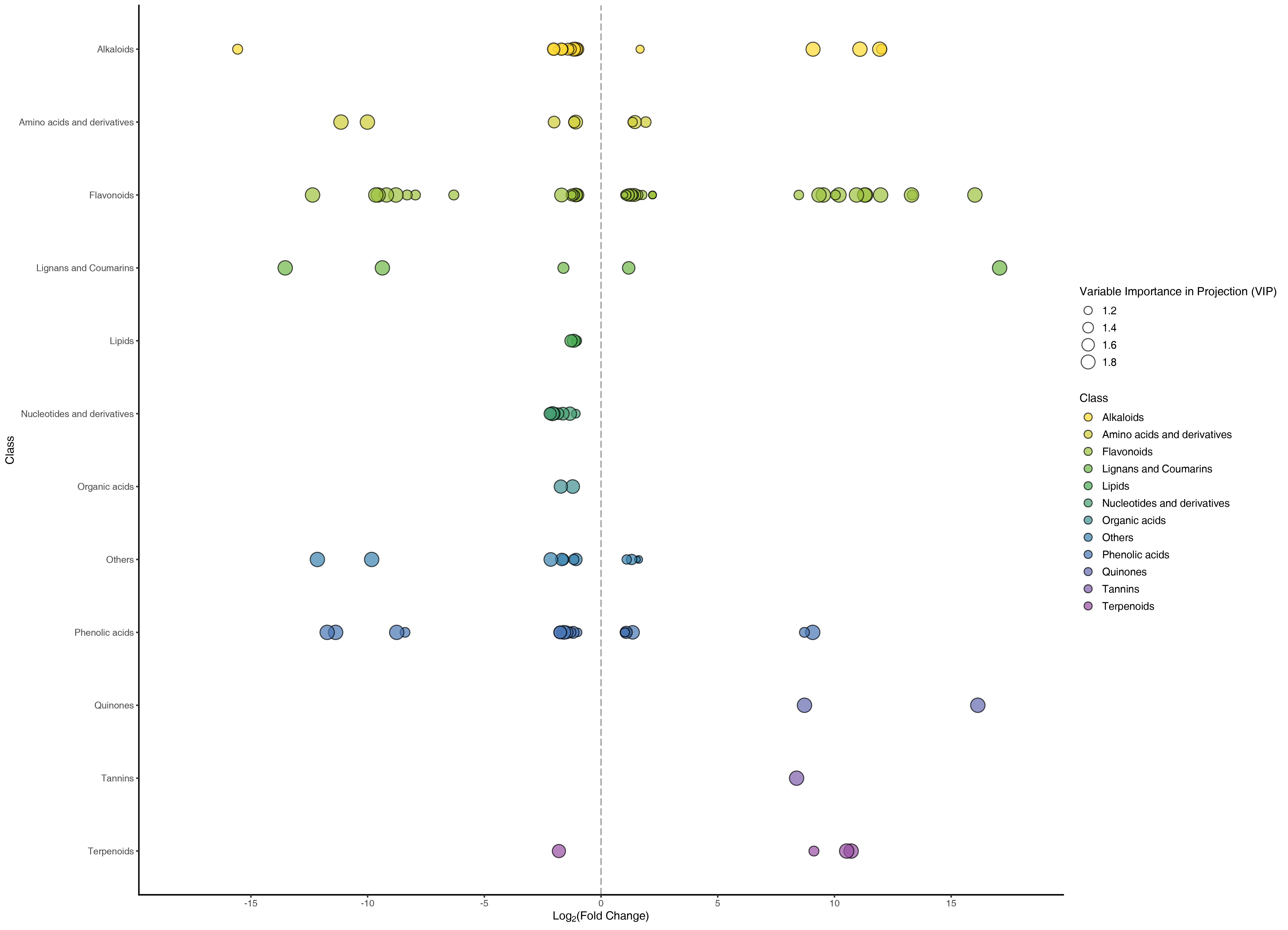
 Three periods of flavonoids Wayne's Chart


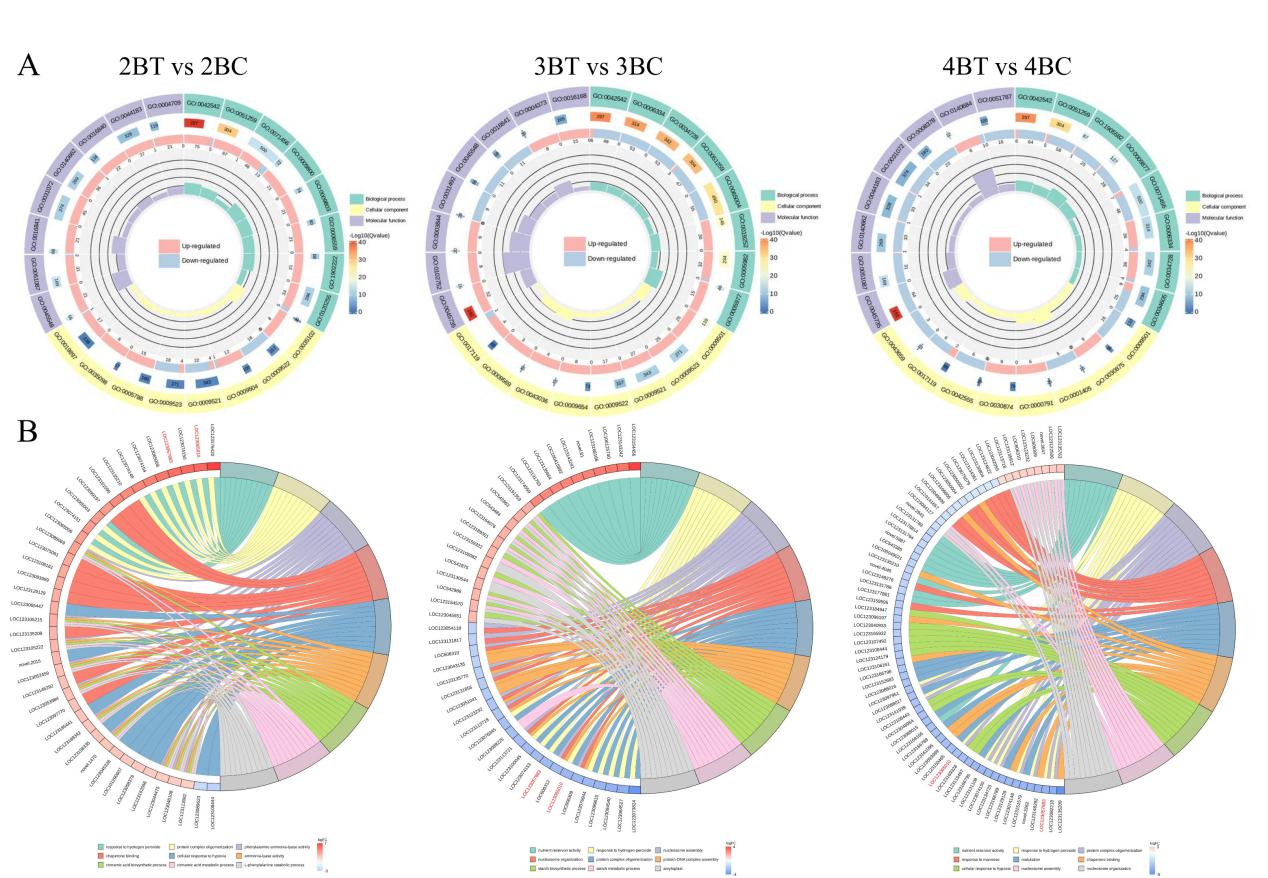


**Fig.S3. Differential gene GO enrichment analysis.** (A) Differential gene GO enrichment circle plot; (B) Differential gene GO enrichment chord plot.
