# Supplementary figures and images for: UV-B induced flavonoid accumulation and related gene expression in blue- grained wheat at different periods of time
Source: Front Plant Sci. 2024 Dec 16;15:1520543. doi: 10.3389/fpls.2024.1520543 (PMC11684391; doi:10.3389/fpls.2024.1520543)

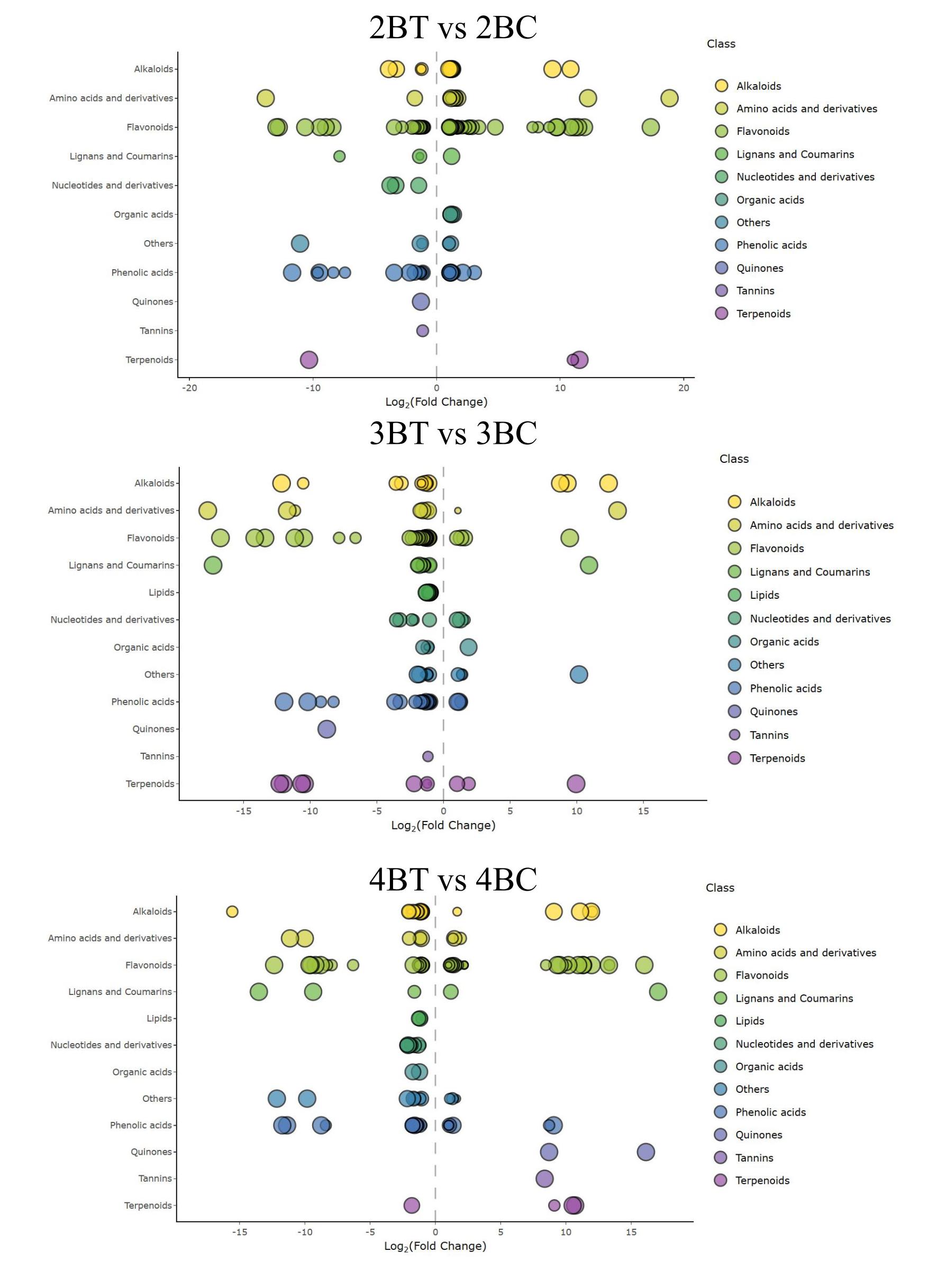

Supplement: Supplementary file 1 [file DataSheet1.zip › supplementary figure/fig.S1.jpg]

## 2BT vs 2BC

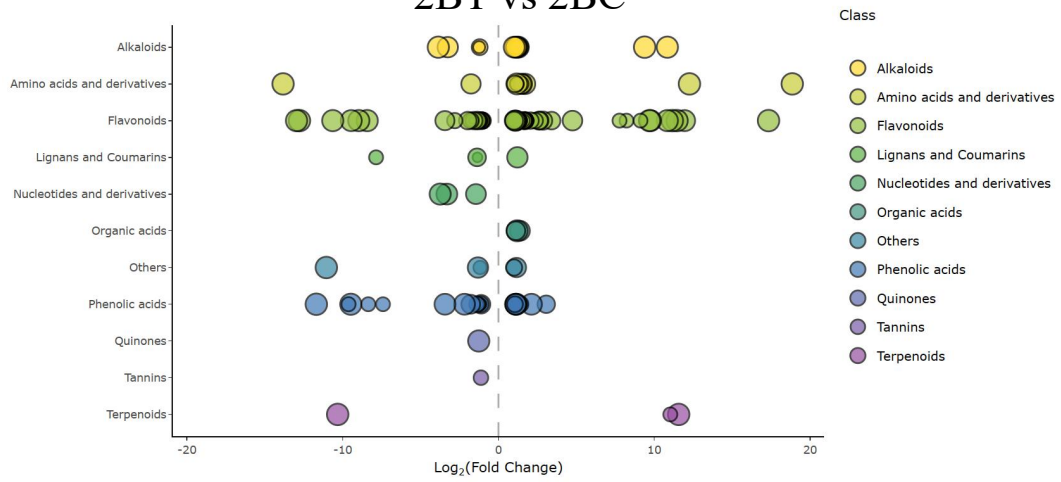

## 3BT vs 3BC

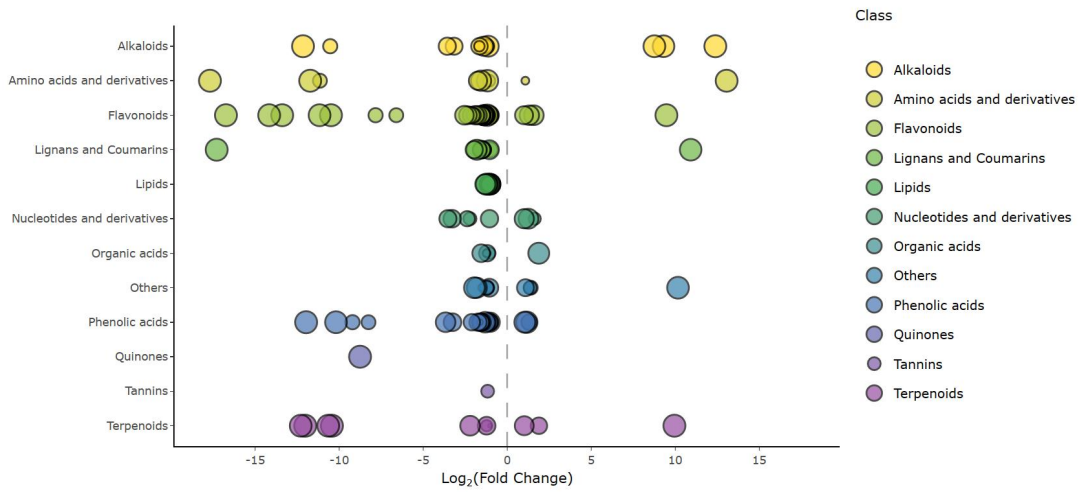

## 4BT vs 4BC

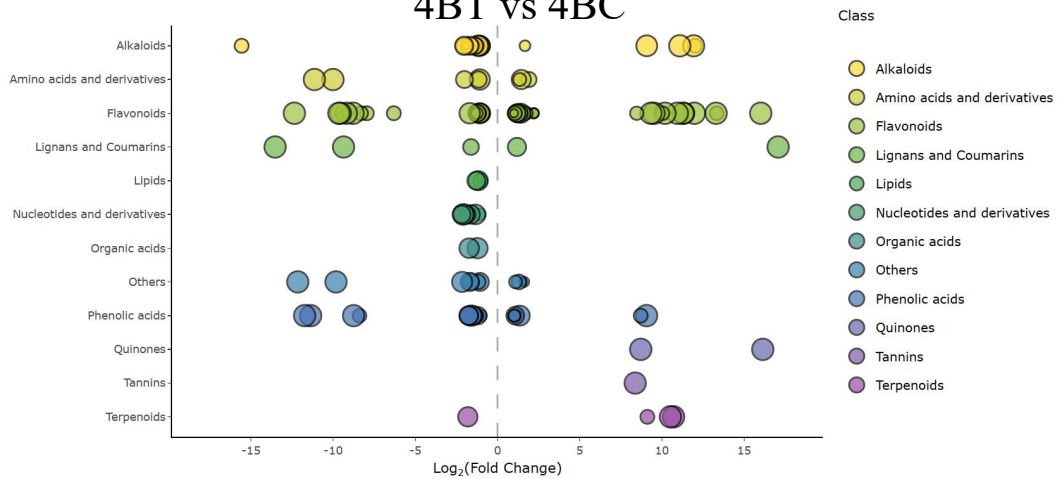

Supplement: Supplementary file 1 [file DataSheet1.zip › supplementary figure/fig.S1.pdf]

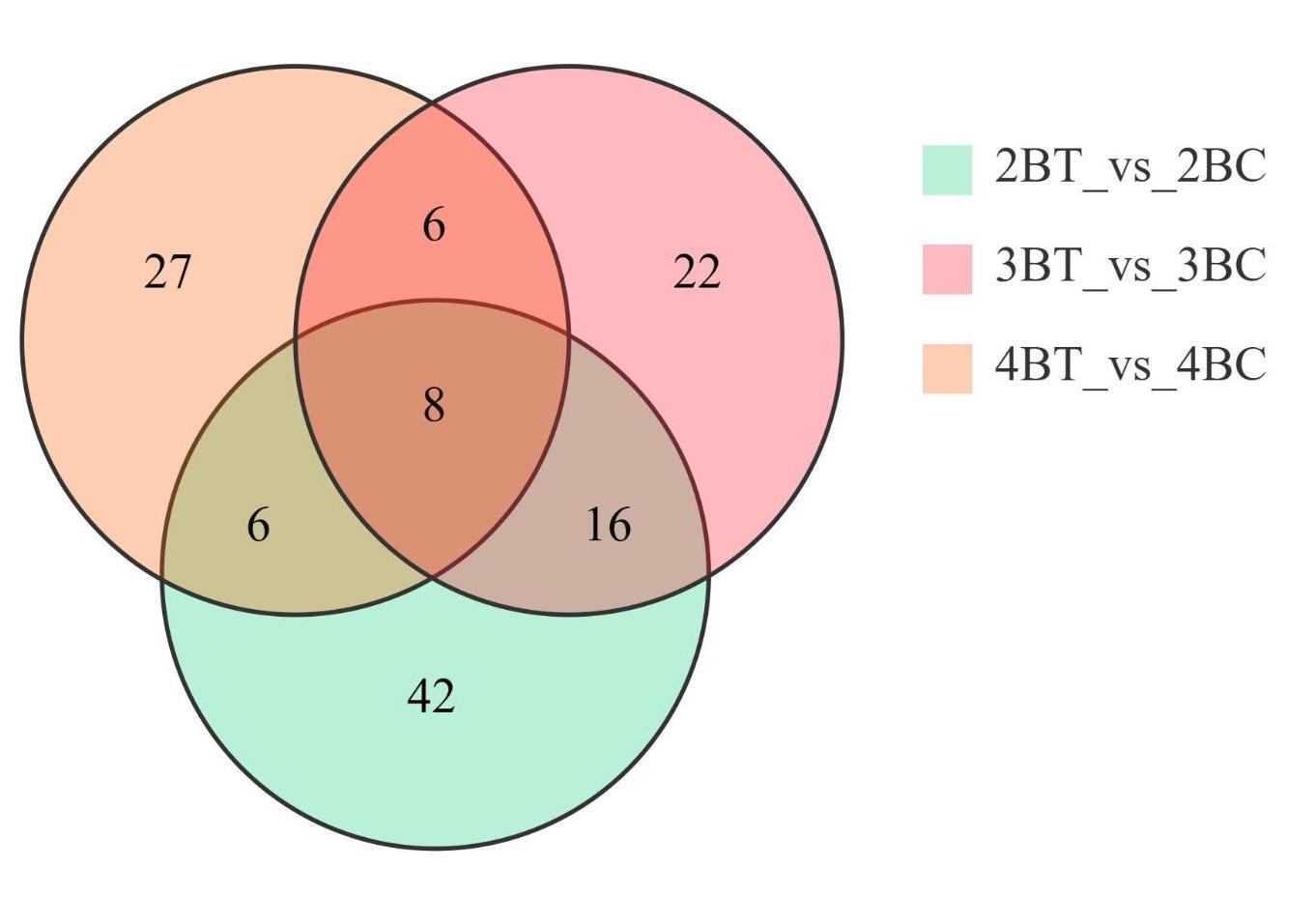

Supplement: Supplementary file 1 [file DataSheet1.zip › supplementary figure/Fig.S2.jpg]

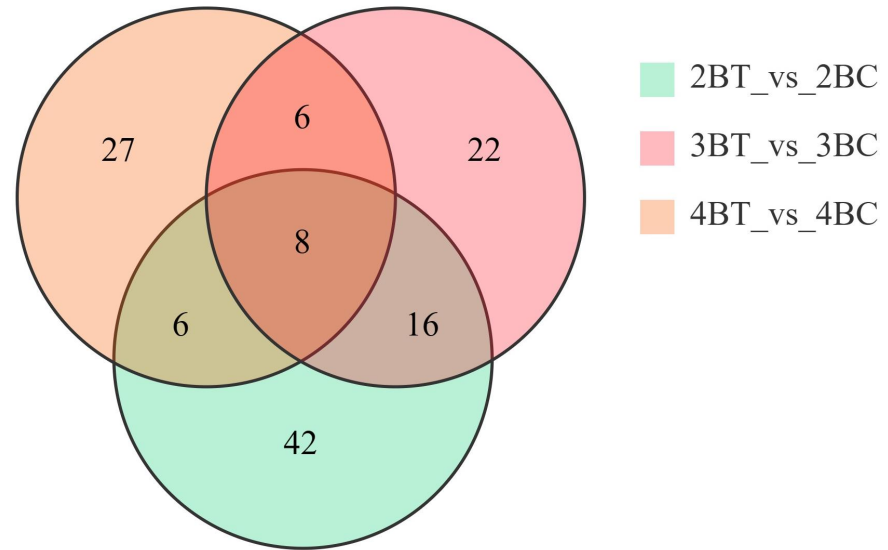

Supplement: Supplementary file 1 [file DataSheet1.zip › supplementary figure/Fig.S2.pdf]

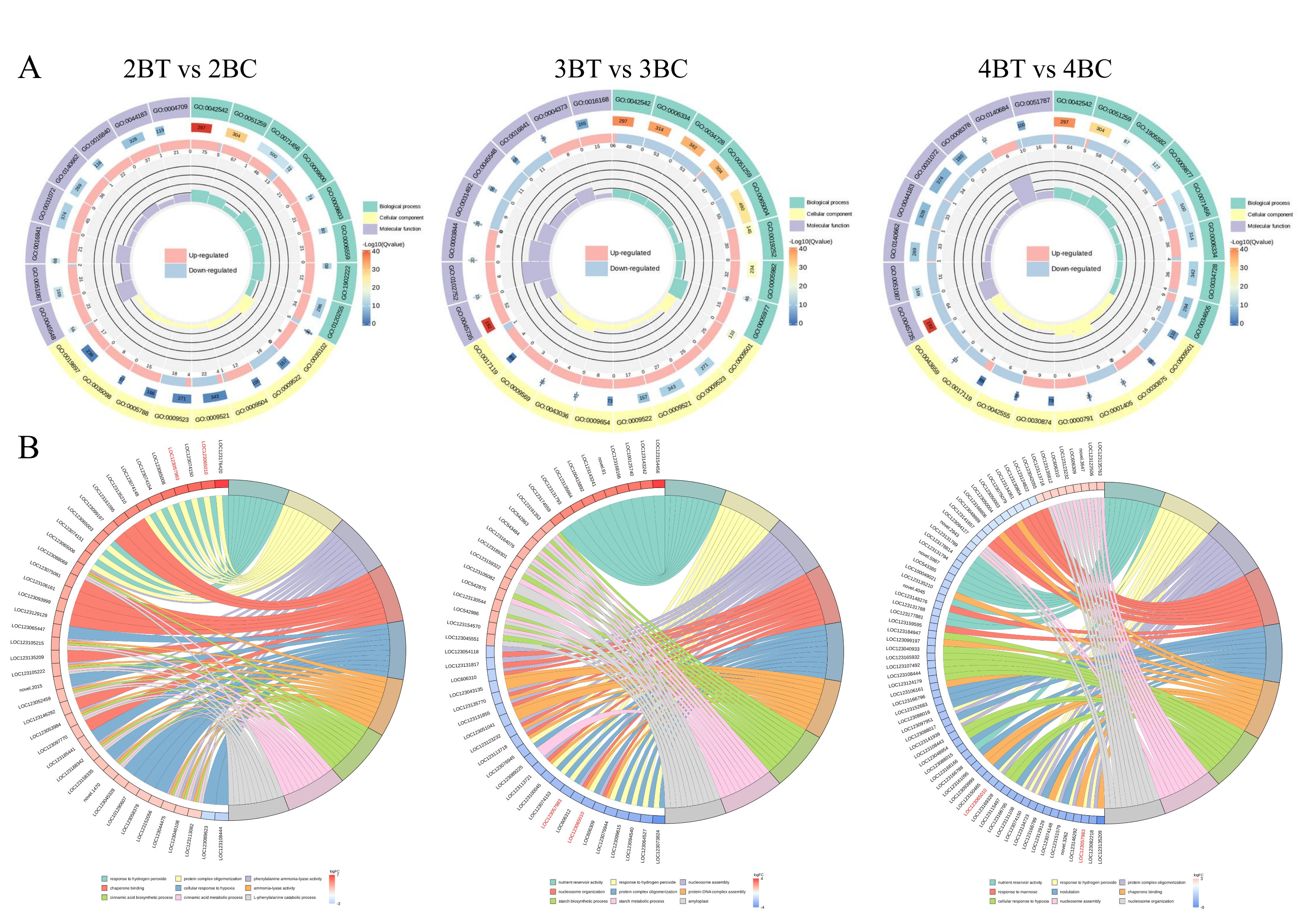

Supplement: Supplementary file 1 [file DataSheet1.zip › supplementary figure/Fig.S3.jpg]

A

2BT vs 2BC

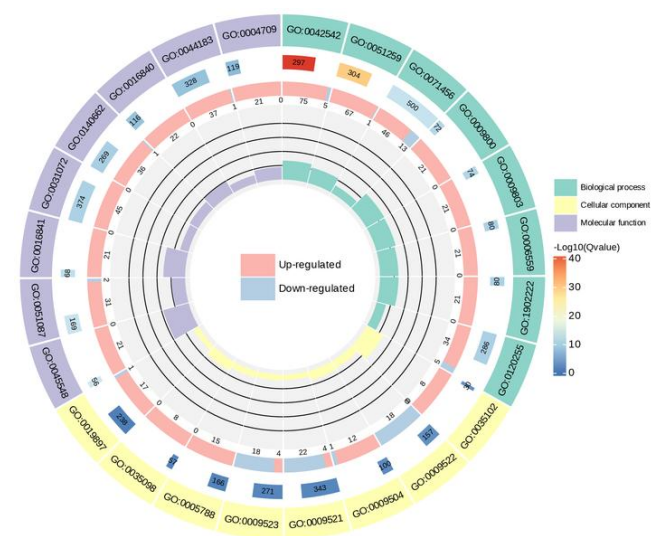

3BT vs 3BC

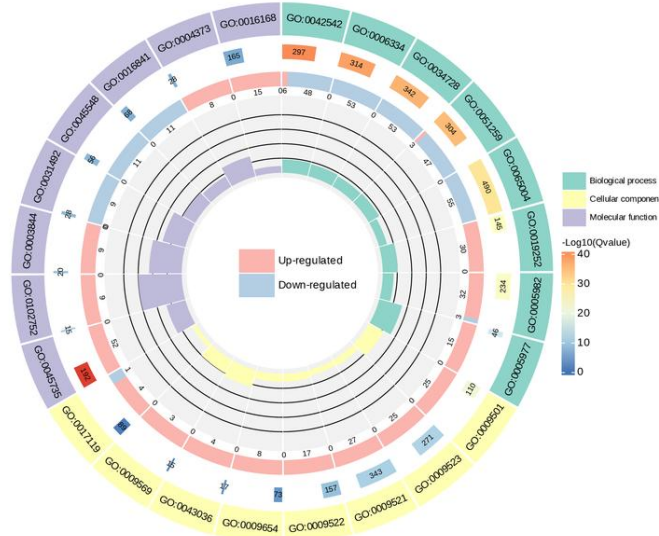

4BT vs 4BC

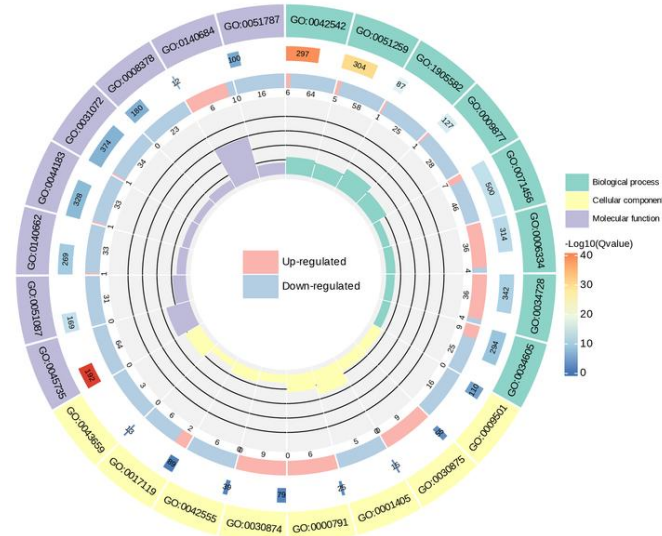

B

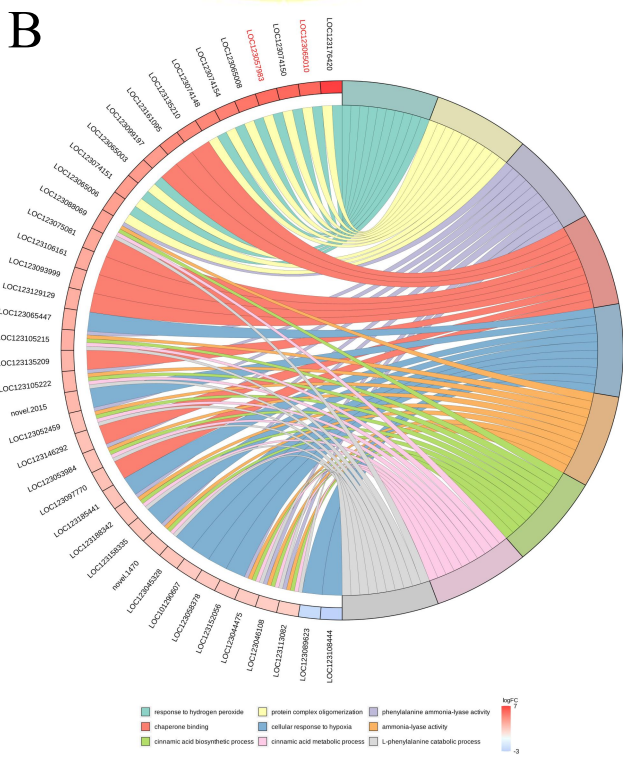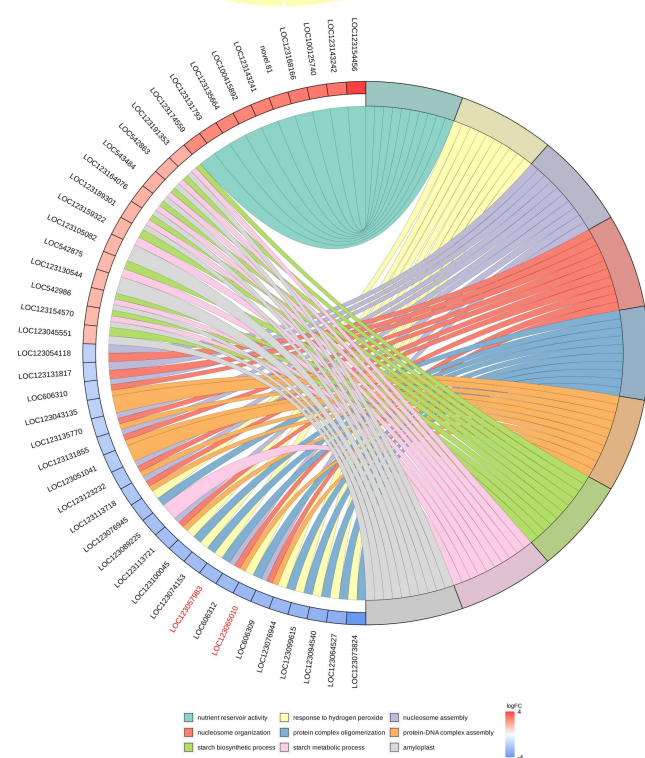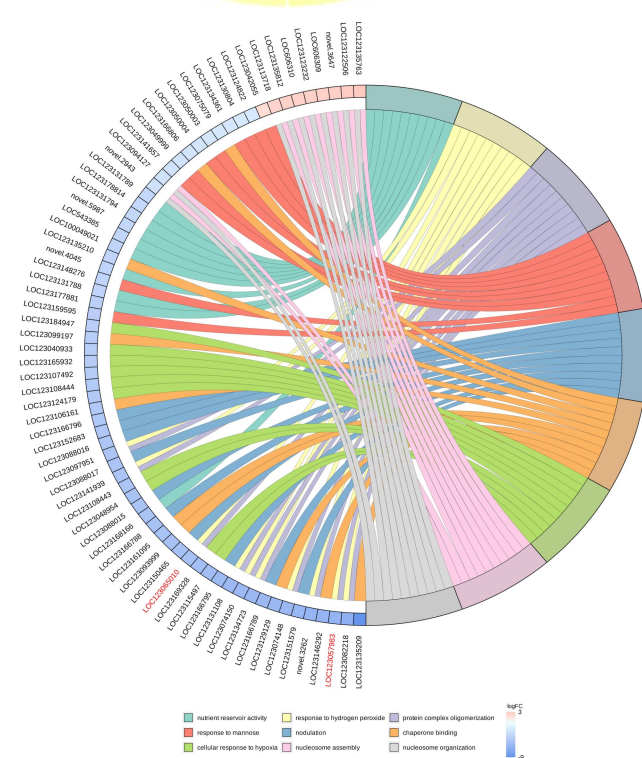

Supplement: Supplementary file 1 [file DataSheet1.zip › supplementary figure/Fig.S3.pdf]
